# Supplementary material for: Is the Thoughts and Health programme feasible in the context of Swedish schools? A quasi-experimental controlled trial study protocol
Source: BMJ Open. 2021 Jan 21;11(1):e040374. doi: 10.1136/bmjopen-2020-040374 (PMC7825266; doi:10.1136/bmjopen-2020-040374)
Supplement: Supplementary data [file bmjopen-2020-040374supp001.pdf]

Information about the study "Thoughts and health" - to prevent mental illness in young people

Background
Mental illness in young people is a growing problem in Sweden and a large part of the world.
Depression is one of the diseases that has the greatest social and economic consequences globally and leads to great human suffering. A first illness often occurs in late adolescence. A first depression increases the risk of relapse.

Preventive efforts to reduce the risk of a first illness should be regarded as an important measure to improve young people's mental health and opportunities for a positive entry into adulthood.

Children with depression at the end of junior high school may risk not being able to finish their compulsory schooling with complete grades, which in turn may affect their future opportunities.

We have now translated and adapted a prevention programme from Icelandic to Swedish. We would like to test whether this programme works in Swedish schools.

In collaboration with Elevhälsan Tjörn Municipality and Orust Municipality and Närhälsan's health centres in the local area and Närhälsan's Research and Development Department, we now want to offer students in these municipalities the opportunity to help us implement the programme and at the same time fill important knowledge gaps in research.

Purpose
To study the effects of an Icelandic prevention programme "Thoughts and health" in Swedish schools.

Request for participation
You are now asked as a student in year 8 on Tjörn for consent to participate in the study and the Thoughts and Health programme.

How is the study done?

All children at the school have been asked to fill out a form with questions about their mental health. All children will receive feedback about the results. Some will receive feedback during their health talk with

the school nurse while others will be offered feedback and assessment by psychologists associated with the project for possible inclusion in the prevention programme. The programme consists of a course involving 12 sessions at the school that are led by a psychologist and the school nurse. The group(s) is put together based on the number of possible participants per school, which means that the composition does not depend upon which class you are in. For you, this means that you will be asked to fill out forms at the start, when half the programme is completed and again at follow-ups. As a participant in the programme, you can of course talk to your parents or guardians about the content of the course, but you should not tell about other participants' experiences. Everyone who is part of the programme or the reference group that is included will be followed up after the end of the programme, at 6, 12 and 18 months. The results from the first questionnaire and the psychologist's assessment are included in the data collected.

Data management and confidentiality

All answers and results will be treated with confidentiality so that unauthorized persons cannot take part in them.
Personal data is registered in accordance with GDPR (The General Data Protection Regulation). The investigator responsible for the study is entrusted with the processing of personal data. According to the GDPR, you have the right to apply for information about which personal data is processed and to demand destruction of the personal data. You and your parents can send your application to the contact person below.

If you want to submit a complaint regarding the processing of personal data, you can contact the Data Inspectorate, which is the supervisory authority for the Data Protection Ordinance. Please note that the Data Protection Officer cannot answer questions concerning specific information about the study but only regarding the application of the Data Protection Regulation.
Contact details of the Data Protection Officer: Anders Larsson, Information Security Coordinator Phone: 070-6552809 anders.h.larsson@vgregion.se
In data processing, your name and personal identity number will be replaced with a code to ensure anonymity. Only those responsible for the study have access to the "code key". Your information will not be passed on. The data will be saved for ten years. The authority responsible for your personal data is primary care Västra Götaland. Lina Kolsmyr tel.
010-441 05 30, narhalsan.dataskyddsombud@vgregion.se Personal data representative Närhälsan, Lillhagsparken 6, 422 50 Hisingsbacka. When the data from the study is compiled, individual individuals will not be able to be identified.

Remuneration
No remuneration will be given for participation.

Volunteering
Your participation is voluntary and you can cancel your participation at any time without giving any reason.

What does the study mean for you and other children in the future?
That you may contribute to the development of student health and that by participating in the research project, you may provide answers to important questions regarding mental health. Your participation will help us to study this prevention programme against depression, the aim of which is to create good routines that in the long run may lead to improved mental health for children and young people.

Persons responsible for the study
If you have any questions, you are welcome to contact us.

Carl Wikberg
PhD, Distriktssjuksköterska
carl.wikberg@allmed.gu.se
0703-821660

Gudny Sveinsdottir
Projektledare, Spec. allmänmedicin
gudny.sveinsdottir@vgregion.se
mobil 0707-980436

Anna Orvefors,
Tf avdelningschef Resurscentrum, Barn- och utbildningsförvaltningen, Tjörns kommun;
anna.orvefors@tjorn.se
tel 0304-601587

Josefine Lilja
FoU Primärvård, Göteborg- och Södra Bohuslän
PhD, Spec. klinisk psykolog, leg. psykoterapeut-KBT, Handledare-KBT
josefine.lilja@vgregion.se
076 - 940 29 69

Consent form - Information about the study "Thoughts and health" - to prevent mental illness in young people

I have been informed, given the opportunity to ask questions, have them answered and agreed to participate in the study and been informed about the right to integrity when the material is published. I have also been informed that my participation in the study is voluntary and that I am right at any time to interrupt my participation in the study without giving a reason and without it affecting my contact with primary care or school.

Print Name of Participant____

Signature of Participant ____

Date _________________________________
Day/month/year

Mailadress____

Mobilenumber____

Print Name of Researcher/person taking the consent______________________________________

Signature of Researcher /person taking the consent_______________________________________

Date _________________________________
Day/month/year
