# Supplementary material for: Is the Thoughts and Health programme feasible in the context of Swedish schools? A quasi-experimental controlled trial study protocol
Source: BMJ Open. 2021 Jan 21;11(1):e040374. doi: 10.1136/bmjopen-2020-040374 (PMC7825266; doi:10.1136/bmjopen-2020-040374)
Supplement: Author's manuscript [file bmjopen-2020-040374.draft_revisions.pdf]

BMJ Open is committed to open peer review. As part of this commitment we make the peer review history of every article we publish publicly available.

When an article is published we post the peer reviewers' comments and the authors' responses online. We also post the versions of the paper that were used during peer review. These are the versions that the peer review comments apply to.

The versions of the paper that follow are the versions that were submitted during the peer review process. They are not the versions of record or the final published versions. They should not be cited or distributed as the published version of this manuscript.

BMJ Open is an open access journal and the full, final, typeset and author-corrected version of record of the manuscript is available on our site with no access controls, subscription charges or pay-per-view fees (http://bmjopen.bmj.com).

If you have any questions on BMJ Open's open peer review process please email
info.bmjopen@bmj.com

BMJ Open

Thoughts and Health – A novel approach to prevent first depression in adolescence in Sweden. A Study Protocol.

Journal:	*BMJ Open*
Manuscript ID	bmjopen-2020-040374
Article Type:	Protocol
Date Submitted by the Author:	13-May-2020
Complete List of Authors:	Wikberg, Carl; University of Gothenburg Institute of Medicine, School of Public Health and Community Medicine<br>Sveinsdottir, Gudny; Vastra Gotaland Primary Health Care, Närhälsan Research and Development Primary Health Care, Region Västra Götaland, Gothenburg, Sweden<br>Augustsson, Pia; University of Gothenburg Department of Public Health and Community Medicine, ;<br>Craighead, Edward; Emory University, Department of Psychology, Psychiatry & Behavioral Sciences<br>Arnarson, Erikur<br>Larsson, Maria; University of Gothenburg Institute of Neuroscience and Physiology, Närhälsan Research and Development Primary Health Care, Region Västra Götaland, Gothenburg, Sweden<br>Marteinsdottir, Ina; University of Kalmar, Department of Medicine and Optometry<br>Lilja, Josefine L.; University of Gothenburg Department of Psychology, Department of Psychology, University of Gothenburg, Gothenburg, Sweden
Keywords:	Child & adolescent psychiatry < PSYCHIATRY, PUBLIC HEALTH, Depression & mood disorders < PSYCHIATRY

SCHOLARONE™
Manuscripts

I, the Submitting Author has the right to grant and does grant on behalf of all authors of the Work (as defined in the below author licence), an exclusive licence and/or a non-exclusive licence for contributions from authors who are: i) UK Crown employees; ii) where BMJ has agreed a CC-BY licence shall apply, and/or iii) in accordance with the terms applicable for US Federal Government officers or employees acting as part of their official duties; on a worldwide, perpetual, irrevocable, royalty-free basis to BMJ Publishing Group Ltd ("BMJ") its licensees and where the relevant Journal is co-owned by BMJ to the co-owners of the Journal, to publish the Work in this journal and any other BMJ products and to exploit all rights, as set out in our licence.

The Submitting Author accepts and understands that any supply made under these terms is made by BMJ to the Submitting Author unless you are acting as an employee on behalf of your employer or a postgraduate student of an affiliated institution which is paying any applicable article publishing charge ("APC") for Open Access articles. Where the Submitting Author wishes to make the Work available on an Open Access basis (and intends to pay the relevant APC), the terms of reuse of such Open Access shall be governed by a Creative Commons licence – details of these licences and which Creative Commons licence will apply to this Work are set out in our licence referred to above.

Other than as permitted in any relevant BMJ Author's Self Archiving Policies, I confirm this Work has not been accepted for publication elsewhere, is not being considered for publication elsewhere and does not duplicate material already published. I confirm all authors consent to publication of this Work and authorise the granting of this licence.

For peer review only

Thoughts and Health – A novel approach to prevent first depression in adolescence in Sweden. A Study Protocol.

Carl Wikberg[1,2], Pia Augustsson[1,2], Gudny Sveinsdottir[2] ,Edward W Craighead[3], Erikur Örn Arnarson[4], Ina Marteinsdottir[5], Maria Larsson[2.6], Josefine L Lilja[2,7].

Affiliation:

1 School of Public Health and Community Medicine, Institute of Medicine, Sahlgrenska Academy, University of Gothenburg, Gothenburg, Sweden.

2 Närhälsan Research and Development Primary Health Care, Region Västra Götaland, Gothenburg, Sweden.

3 Department of Psychiatry and Behavioral Sciences and the Department of Psychology, Emory University, 12 Executive Park Drive NE, Suite 200, Atlanta, GA 30329, USA.

4 Department of Psychiatry, Landspítali-University Hospital, Reykjavík, Iceland.

5 Department of Clinical and Experimental Medicine, Center for Social and Affective Neuroscience, Linköping University, 581 85, Linköping, Sweden.

6 Institute of Neuroscience and Physiology, Department of Health and Rehabilitation, Unit of Physiotherapy, The Sahlgrenska Academy at University of Gothenburg, Gothenburg, Sweden.

7 Department of Psychology, University of Gothenburg, Gothenburg, Sweden.

Abstract

Introduction; Clinical depression is a substantial problem among adolescents, increasing significantly at about age 15. It causes impairment in social, academic, and familial relationships as well as ongoing cognitive and emotional difficulties for the individual. A study in Iceland demonstrated that a cognitive-behavioural, developmentally based intervention programme, "Thoughts and Health", prevented initial episodes of depression and/or dysthymia (MDD/DYS) in adolescents for up to 12 months following completion of the programme. We would like to test the feasibility of implementing the Icelandic method in a Swedish context and to evaluate the long-term effects of such a programme.

Methods and analysis; A quasi-experimental controlled design combined with qualitative and quantitative methods will be used to address the research questions.

Depending on type of variable, baseline comparisons of the two groups of relevant initial measures will be evaluated with t-tests or chi-square analyses. The effects of the programme on the development of depressive symptoms will be evaluated for the 6-, 12-, and 18-month data using the Cox proportional hazards model to estimate survival curves and rates for different thresholds of combined scores of depressive symptoms. Index parental depression at baseline will be tested as a moderator in the evaluation of the prevention programme effects.

Ethics and dissemination.

This study is approved by the Swedish Ethical Review Board (reference number 2019-03347) in Gothenburg.

For peer review only

We plan to disseminate the knowledge gained from this study by publishing our results in peer-reviewed scientific journals, giving presentations at national and international conferences and spreading information through local and national media channels. We will approach local and national politicians to ensure prolongment of the project.

Registration details: NCT04128644 Clinical trials.

Strengths and limitations of this study

- Quasi-experimental controlled design will provide information about whether the intervention prevents depressive episodes in adolescence in a Swedish sample.
- Qualitative data will shed light on how the intervention was perceived by the adolescents and how the personnel experienced the implementation process.
- Quantitative data will provide information regarding adolescents' mental health and social functioning.
- Follow-up assessments will provide information regarding the intervention's long-term effects on school performance and mental health.
- The study lacks a randomized controlled design and has few participants, which limits its generalisability.

NCT04128644 Clinical trials

Keywords: Depression, Adolescence, Prevention, Feasibility

Word count: 3510

Correspondence to

Carl Wikberg – carl.wikberg@allmed.gu.se

+46703821660

Introduction

Clinical depression is a substantial problem among adolescents, increasing significantly at about age 15. It causes impairment in social, academic, and familial relationships as well as ongoing cognitive and emotional difficulties for the individual [1, 2].

A study in Iceland demonstrated that a cognitive-behavioural, developmentally based intervention programme called "Thoughts and Health" prevented initial episodes of depression and/or dysthymia (MDD/DYS) in adolescents for up to 12 months following completion of the programme [3,4]. Other studies have demonstrated that similar programmes can be employed to prevent depression among teens who have never been depressed and/or who previously have been depressed [5–8].

However, despite the positive impact of such programmes, preventive effects have not been consistently obtained [9–11]. Reviews have concluded that universal prevention programmes (programmes applied to the general population) have not been effective in preventing depression (see [12, 13]), and excellently conducted studies in Australia have provided data consistent with that conclusion [14, 15]. In general, only targeted programmes (programmes aimed at selective–for populations exposed to risk factors or indicated–for populations who are experiencing some symptoms) have shown significant short-term effects in preventing depression (e.g., [1, 5–7]).

For peer review only

Most programmes aimed at preventing depression have included both youth who have suffered a prior depression and those who have never been depressed. The study of prevention of relapse or recurrence in prevention programmes for those who have already suffered a major depressive disorder (MDD) is clearly useful, but earlier studies have not distinguished between prevention of the first episode of MDD and the prevention of relapse/recurrence of MDD. However, because of the recurrent nature of the disorder and the differing causal factors and probability patterns of first versus subsequent episodes [16] and in order to prevent the deleterious effects of an episode of MDD in youth, it is especially important to study the prevention of the first or initial episode among never-previously-depressed subjects.

Since clinical depression among adolescents increases significantly at about age 15, and Swedish children finish their compulsory school (junior high school) at that age, being at risk for depression can be a reason for failing their final examinations, thereby limiting their chances for further academic studies.

We would like to test the feasibility of implementing the Icelandic method in a Swedish context and to evaluate the long-term effects of such a programme.

This is, to our knowledge, the first study in Sweden that employs a longitudinal design to evaluate the effects of a school programme aimed at preventing depression in adolescents.

This study will be implemented together with the municipalities of Tjörn and Orust, two islands off the west coast of Sweden, and their four middle schools. Thus, this study is built on a close collaboration between academia, primary care, and the community.

We are hopeful that this programme will be transferrable to a Swedish context and that it will be possible to further develop and enhance the method.

We hypothesise that programme implementation will reduce the risk of developing depressive symptoms among participating students, and we will examine this in participating adolescents up to 18 months after programme completion. We also hypothesise that adolescents in the intervention group who participate in the programme will finish school with complete grades to a higher extent than the control group.

Aim

This study aims to test the feasibility of implementing an Icelandic cognitive-behavioural programme designed to prevent depression, "Thoughts and Health" [3,4], in a Swedish school setting.

The perspectives of both the participating adolescents and the primary care professionals who lead and support the programme in the schools will be taken into account when evaluating the programme's feasibility.

The study also aims to evaluate the number of students who finish junior high school (ninth grade) with complete grades. In this study, the outcomes concerning complete grades will be compared between the students participating in the intervention programme and those in the control group.

Our primary research questions are:

Is the "Thoughts and Health" programme feasible in the context of Swedish schools, from the perspective of the students participating in the programme?

Is the "Thoughts and Health" programme feasible in the context of Swedish schools, from the perspective of the primary care professionals leading and supporting the programme implementation?

Our secondary research questions are:

For peer review only

Does participation in the “Thoughts and Health” programme affect the number of students developing depressive symptoms?

Will the participation in the course “Thoughts and Health” increase the participants' chances of completing compulsory school with high school eligibility?

Method

Study design

A quasi-experimental controlled design combined with qualitative and quantitative methods will be used to address the research questions. NCT04128644 , Clinicaltrials.com

We have used the SPIRITreporting guidelines [17]

Participants

Four junior high schools from the municipality of Tjörn (Häggvall and Bleket, intervention arm) and Orust (Henån och Ängås, control arm) will participate in the study. These schools were chosen on the basis of their socioeconomic similarity, willingness to participate and available staff. Two intervention courses are planned (I and II), with course I beginning at the start of the fall semester 2019 and course II beginning at the start of the fall semester 2020.

In total, approximately 320 eighth grade Swedish adolescents aged 14 years will participate in a pre-intervention screening process per course. In the pre-intervention screening process, all students will fill out the Children’s Depression Inventory (CDI, described below) [18] assessment instrument via an online questionnaire tool. This will be part of the existing “Student Health” ordinary assignments that include health and mental health status checkups as per the Swedish School Law (Skollagen 2010:800).

All students who score above the 75th percentile on the CDI screening will be qualified initially for possible inclusion in the study (expected n=80 total per course). They will receive an invitation together with their parents to attend a psychological assessment interview (the Mini International Neuropsychiatric Interview for Children and Adolescents ((MINI-KID), described below) [19]. At the interview, they will be given the opportunity to discuss their CDI result and if needed, to be channelled to the right level of care. (Example: If a student scored very high on CDI, the psychologist will need to decide whether there is any underlying diagnosis that requires treatment or if the student has any other condition that also requires immediate attention and treatment.) If a student is diagnosed with depression at this phase, he or she will no longer be eligible to participate in the study and will be directed towards appropriate mental health care resources in the community. Prior to attending this psychological assessment, students and their parents will fill out the Revised Children’s Anxiety and Depression Scale (RCADS) [20] assessment instrument.

Based on the MINI-KID results, students who meet criteria for having any current or past major depression, dysthymia, bipolar disorder I or II, cyclothymia, anorexia, bulimia, psychotic disorder, alcohol or substance dependence, attention deficit hyperactivity disorder (ADHD), oppositional defiant disorder (ODD), or conduct disorder will be excluded. All other students thus fulfil inclusion criteria. All students, both in the intervention and control arms, will be invited to participate in the study via a letter (expected n=40 total per study arm). See table 1 for inclusion and exclusion criteria.

Thus, about 20 students per course will be offered to participate in the intervention programme. These students will be approached by the programme staff and offered the opportunity to participate in the baseline clinical interview and assessment, the intervention programme, and post-intervention follow-up assessments at 1, 6, 12, and 18 months after programme completion (figure 1).

For peer review only

Participants in the intervention arm will be offered tickets to the cinema as a gesture of gratitude for their participation in each of the follow-up assessments.

Measures and assessments

Measures and assessments are described below. The time point for each measure or assessment is shown in table 2. Outcome Measures Over Time.

CDI, ordinal variables (collected during pre-intervention phase, at course start, and at 6-, 12-, and 18-months follow-up). The CDI is a 27-item self-reported, symptom-oriented scale that rates the severity of depression during the past month. It is designed for children/adolescents, ages 7-17 years. Each of the 27 items comprises 3 sentences used by the student to rate the severity of the symptoms. This inventory has previously shown excellent internal consistency (alpha coefficients of .83 to .94). The present study utilises the total score of the Swedish version CDI total score.

Parental Depression, binary variable (collected during pre-intervention phase). At the time of the baseline interview, adolescents will be asked if their parents previously have suffered from or currently suffer from depression. Similarly, parents will be asked if they have previously suffered from or currently suffer from depression.

MINI-KID, categorical variables (collected during pre-intervention phase and at 6-, 12-, and 18-months follow-up). The MINI-KID is a structured interview for children and young people aged 4-17 years old. It is based on diagnoses using the Diagnostic and Statistical Manual of Mental Disorders (DSM-5)[21] and the tenth edition of International Statistical Classification of Diseases and Related Health Problems (ICD-10). Both parents and youths will participate in the interview, but the interview can also be conducted with only young people. The interview aims to ensure diagnosis and inclusion/exclusion criteria.

Children's Global Assessment Scale (CGAS)[22], ordinal variables (collected during pre-intervention phase). The CGAS will be used to measure global functioning. The assessment will be made by the person who conducts the MINI-KID interview. The CGAS estimate is made on a scale of 1-100, where a higher score indicates a better functioning of life / higher functional level.

The Revised Children's Anxiety and Depression Scale (RCADS), ordinal variables (collected during pre-intervention phase and at 6-, 12-, and 18-months follow-up). RCADS is a widely used questionnaire designed to assess clinical syndromes of anxiety as well as depression, building on the DSM-IV criteria. The RCADS provides two total scores, and 6 subscales: Separation Anxiety Disorder (SAD), Social Phobia (SoP), Obsessive Compulsive Disorder (OCD), Panic Disorder (PD), Generalized Anxiety Disorder (GAD), and MDD. The internal consistency of the RCADS subscales is high, with Cronbach alphas ranging from 0.78 to 0.88. There are two versions of RCADS; one for adolescents (RCADS) and one for parents (RCADS-P).

Demographic information and somatic health, categorical variables (collected at course start and at 6-, 12-, and 18-months follow-up). The baseline questionnaire will include items regarding demographics, physical activity, and drug use, and some questions about mobile and internet usage.

Education Work and Social Adjustment Scale (EWSAS) [23], ordinal variables (collected at course start, course middle (6 weeks after course start), and at 6-, 12-, and 18-months follow-up). The EWSAS measures general level of function. It is an adaptation of the Work and Social adjustment Scale (WSAS) and measures the level of function in five areas: relationships, family, leisure time, social activities, and school and everyday activities.

For peer review only

Mental Health Continuum – Short Form (MHC – SF), ordinal variables (collected at course start, course middle (6 weeks after course start), and at 6-, 12-, and 18-months follow-up). The MHC-SF is an increasingly used questionnaire intending to measure three components of well-being: emotional, social, and psychological. It has shown excellent internal consistency (>.80) and discriminant validity in adolescents (ages 12-18 years) and adults in the U.S., in the Netherlands, and in South Africa [24-27]. MHC-SF consists of 14 items asking about the frequency of well-being experiences during the last month. There are three items concerning emotional well-being such as feelings of being happy, interested in life and satisfied, five items concerning social well-being referring to acceptance, actualisation, contribution, coherence and integration and six items concerning psychological well-being capturing purpose in life, environmental mastery, autonomy, personal growth, positive relations and self-acceptance. Answers are given on a 6-point Likert scale from 0 = never, 1 = once or twice, 2 = about once a week, 3 = about 2 or 3 times a week, 4 = almost every day, 5 = every day. Cronbach's alpha was 0.92 for emotional well-being, 0.88 for social well-being and 0.92 for psychological well-being. Scores on each well-being component are summed up and categorised as "flourishing", "moderately healthy" or "languishing" according to official MHC-SF guidelines. The MHC-SF has been useful in improving the scientific understanding of the risk of future mental illness (major depressive episode, GAD, panic attacks, risk of premature mortality, healthcare utilisation, missed days of work, suicidality, and self-reported academic impairment among college students).

Course surveys, ordinal variables (collected after each course session). After each course session (total of 12 sessions per course), the students as well as primary care professionals leading and supporting the course will directly provide their feedback about the course session by answering a short survey. Different surveys will be provided to students and the professionals, respectively.

Course attendance and dropouts, binary variables (collected during each course session). Attendance and dropouts will be recorded at each course meeting, as a measure of the feasibility and acceptance of the programme.

Focus groups and interviews, qualitative analysis (collected within 1 month after course end and at 18-months follow-up). All participants (students and professionals) will be invited to take part in focus group discussions or individual interviews after the completion of the full course. These discussions will be held separately, with students in one or two groups and the professionals in another, or as individual interviews.

Final junior high grades, categorical variables (collected at approximately 18 months after course end). Students' final grades in junior high school (that is, in ninth grade, or 18 months after their inclusion in the programme) will be collected to be able to evaluate the number of students who finish school with complete grades. The information will be provided by the school administration upon consent from the students.

Data management

A research journal follows the person during the program and follow-up period. The research journal will be kept at the primary care R&D unit, Kungsgatan, in Gothenburg, where data from the research journal is entered. Data in the database at the research unit will only be entered with code numbers. Only the research leaders will then have access to the code key, which is stored in locked (in safes) at the research unit. The research journals are kept anonymous at the research unit during the project evaluation phase. The composition of the research group may change during the course of the project, but only authorized persons who are part of the research group will have access to data. The usual privacy is maintained.

For peer review only

Intervention: Prevention Programme

The prevention programme will be based on the Icelandic "Thought and Feelings" programme [1] and will include 12 group sessions of 90-minutes duration, delivered twice per week for two weeks and then once a week for eight weeks in the school setting by "Student Health" school psychologists. School nurses will also attend each session as support for the students. The programme uses two intervention manuals: a group leader manual and a student manual. Both of these manuals have been translated first from Icelandic to English, and then from the English version into Swedish, and finally corrected for language in the Swedish version. After translation, the manuals were additionally reviewed by several eighth-grade students for cultural acceptability (e.g., word choice) and adapted as per their feedback. The course material is based on cognitive behaviour theory, and the course will be held as a group activity. The course was originally developed as a group activity, and we will test this method in our study, as part of the evaluation of whether the method is suitable for the adolescents of today. The course contains group sessions with a specific subject, after which they will describe their state of health and feelings in a diary and receive some home assignments to be completed between sessions. All sessions start with a light refreshment and a résumé of the last session. Session #1 is an introduction. Session #2 is about "the spiral", that is, how negative or positive thoughts and actions affect subsequent actions and feelings. Session #3 is about relaxation. Session #4 will discuss the concept of a "baseline". The remaining sessions deal with the following topics: setting targets (#5), feelings (#6), negative thoughts (#7), irrational thoughts (#8), self-perception (#9), communication (#10), stop and reconsideration (#11) and consummation (#12, final session).

Statistical Analysis

Depending on type of variable, baseline comparisons of the two groups of relevant initial measures will be evaluated with t-tests or chi-square analyses. The effects of the program on the development of depressive symptoms will be evaluated for the 6-, 12-, and 18-month data using the Cox proportional hazards model to estimate survival curves and rates for different thresholds of combined scores of depressive symptoms. Index parental depression at baseline will be tested as a moderator in the evaluation of the prevention programme effects. Even though the sample in this part of the project is rather small, we will stratify all results by different variables in the baseline questionnaire including gender, socioeconomic status and mobile/internet use, among others.

The qualitative data collected through interviews and focus group discussions will not require any statistical analysis but will be analysed using qualitative analytical methods such as thematic analysis.

Ethics

This study is approved by the Swedish Ethical Review Board (reference number 2019-03347) in Gothenburg. Since participants are adolescents <15 years, consent from both parents and the adolescents themselves will be obtained, as required by applicable laws and regulations.

This study is based on a validated programme that has been conducted in other countries showing good preventive effect. Based on previous studies, we expect preventive effects of the intervention in the participating adolescents. To ensure that the risks are minimal throughout programme implementation and follow-up, we will provide all participants from the pre-intervention phase through post-intervention follow-up, in both control and intervention arms, with professional help in the form of psychologists, doctors and nurses if needed. The research group has solid experience implementing the treatment programme in other settings; their experience indicates that the intervention is perceived as positive by students and that participation in the group work has not been stigmatising. All measures and assessments used in the study are validated and tested in many other studies. All researchers and professionals in this study have good paediatric psychiatric competence.

For peer review only

They are used to talking with adolescents and will handle unpredictable situations carefully. All personnel involved in the study receive training in the intervention. The research team is trained in good clinical practice (GCP). The study subjects will never be alone during the intervention, as the intervention is carried out in groups and by trained staff. We will invoke the privacy of group members and all relevant legislation will be followed regarding use of research data.

Results from the study will be presented so that no individuals, including students, parents, and the employer representatives, will be identifiable. All results will be presented on a group level. All participation is voluntary, and the student can withdraw from the study at any time.

The trial will be conducted in compliance with this study protocol, the Declaration of Helsinki and Good Clinical Practice (GCP).

Plans for dissemination of the study outcome

We plan to disseminate the knowledge gained from this study by publishing our results in peer-reviewed scientific journals, giving presentations at national and international conferences, and spreading information through local and national media channels. We will approach local and national politicians to ensure prolongment of the project.

Discussion

The study protocol of this research project has shown how the planning and organisation of the research make it feasible to answer the four research questions.

Question (1). Is the “Thoughts and Health” programme feasible in the context of Swedish schools, from the perspective of the students participating in the programme? The answer will be based on the qualitative interviews and focus group discussions conducted with adolescents participating in the intervention group.

Question (2). Is the “Thoughts and Health” programme feasible in the context of Swedish schools, from the perspective of the primary care professionals leading and supporting the programme implementation? The answer will be based on the analysis of interviews with the professionals who have taken part in focus group discussions or individual interviews after the completion of the intervention course.

Question (3). Does participation in the “Thoughts and Health” programme affect the number of students developing depressive symptoms? The answer will be based on before and after measurements, with child and adult reports and statistical analyses. Comparisons of effect sizes will be made between the arms of the study (control schools and intervention schools).

Question (4). Will the participation in the course “Thoughts and Health” increase the participants' chances of completing middle school with high school eligibility? The answer will be based on follow-up measurements of completion and final grades at graduation from middle school. Comparisons of effect sizes will be made between the arms of the study (control schools and intervention schools).

For peer review only

Current trial status

Recruitment of participants started in September 2019, and the last participant is expected to reach the primary endpoint (6-month follow-up) in November 2020. Primary data analysis will begin in May 2020. The naturalistic follow-up phase of the trial will continue until September 2021.

Patient and public involvement

We will receive input from participants and professionals from focus group discussion and interviews, which will guide the continuation   of the intervention study. In the current trial, no participants were involved in the design of the study or in the decision of outcome measures.

Members from the public or students were not involved in designing this study.

We will assess the trial interventions on the participants by collecting information through e-mailed self-rating questionnaire at the end of programme.

For peer review only

Table 1. *Inclusion and exclusion criteria*

Inclusion criteria	Exclusion criteria
**Eighth grader**	**Ongoing or past depression**
Guardian of eighth grader	Addiction
**Understand and be able to fill out**	**Past or current suicidal attempt or ideation**
**questionnaires independently**	Neuropsychiatric diagnosis that precludes group activities
Completed CDI and be above 75e percentile	**Not high enough level of function on the CGAS**
**Completed psychological assessment**	Lacking sufficient Swedish language skills, written and oral
Written and oral consent	**Serious mental illness**
	Ongoing psychotherapeutic treatment

Table 2. *Instruments and follow-up assessments*

	**Screening**	**Pre Intervention**	**During treatment**	**Post-treatment**	**6-month follow-up**	**12-month f[illegible] up**
**Clinician-rated instruments**			**6 weeks**	**3 months**		
MINI-KID		x		x	x	x
CGAS		x		x	x	x
CGI				x	x	x
**Self-rated intsruments**						
CDI	x	x	x	x	x	x
RCADS		x	x	x	x	x
EWSAS		x	x	x	x	x
MHC-SF		x	x	x	x	x
Course surveys			x	x	x	x
Baseline Questionnaire		x		x	x	x
**Qualitative method**						
Focus group discussions				x		
Individual interviews				x		

Acknowledgement

We would like to thank participating schools. We would like to acknowledge the people who are participating in making this project real. Project manager and GP Gudny Sveinsdottir, School Nurses Helena Nordevik Pedersen, Gunilla Börjesson and Inger Höber, Psychologists Camilla Johansson and Magnus Hjorth, Head of Primary care centre in Tjörn Ann-Sofie Lekander, GPs Torbjörn Erneholm and Head of School Health in Tjörn Anna Orvefors. Thanks to Elizabeth Cantor-Graae for language editing. Thanks to Jenna Anderson from Grants Office (Gothia Forum) for help in funding process.

Funding

Social investment grants from Region Västra Götaland, Sweden. Granted: RUN 2018-00614.

Declaration of interest

The author(s) of this paper declare(s) that there is no conflict of interest.

Contributorship statement

The authors have contributed, are contributing and will contribute to this study, its conductance and publication of study results as follows: Substantial contributions to the conception or design of the work: CW, GS, WEC, EÖA, and JL. Statistics: ML. Planning, conduct and reporting of the work: CW, GS, PA and JL. Drafting the manuscript: CW and JL. Critical revision of the article: all declared authors. Final approval of the version published: all declared authors. Agreement to be accountable for all aspects of the work are resolved: all declared author

For peer review only

References

1. Hankin BL, Abramson LY, Moffitt TE, *et al.* Development of depression from preadolescence to young adulthood: emerging gender differences in a 10-year longitudinal study. *J Abnorm Psychol* 1998;107:128–40. doi:10.1037//0021-843x.107.1.128

2. Lewinsohn PM, Rohde P, Klein DN, *et al.* Natural course of adolescent major depressive disorder: I. Continuity into young adulthood. *J Am Acad Child Adolesc Psychiatry* 1999;38:56–63. doi:10.1097/00004583-199901000-00020

3. Arnarson EO, Craighead WE. Prevention of depression among Icelandic adolescents. *Behav Res Ther* 2009;47:577–85. doi:10.1016/j.brat.2009.03.011

4. Arnarson EO, Craighead WE. Prevention of depression among Icelandic adolescents: a 12-month follow-up. *Behav Res Ther* 2011;49:170–4. doi:10.1016/j.brat.2010.12.008

5. Services S beredning för medicinsk och social utvärdering (SBU); SA for HTA and A of S. Program för att förebygga psykisk ohälsa hos barn. SBU 2010. https://www.sbu.se/sv/publikationer/SBU-utvarderar/program-for-att-forebygga-psykisk-ohalsa-hos-barn/ (accessed 3 Feb 2020).

6. Garber J, Clarke GN, Weersing VR, *et al.* Prevention of depression in at-risk adolescents: a randomized controlled trial. *JAMA* 2009;301:2215–24. doi:10.1001/jama.2009.788

7. Stice E, Rohde P, Seeley JR, *et al.* Brief Cognitive-Behavioral Depression Prevention Program for High-Risk Adolescents Outperforms Two Alternative Interventions: A Randomized Efficacy Trial. *J Consult Clin Psychol* 2008;76:595. doi:10.1037/a0012645

8. Clarke GN, Hawkins W, Murphy M, *et al.* Targeted prevention of unipolar depressive disorder in an at-risk sample of high school adolescents: a randomized trial of a group cognitive intervention. *J Am Acad Child Adolesc Psychiatry* 1995;34:312–21. doi:10.1097/00004583-199503000-00016

9. Gillham JE, Hamilton J, Freres DR, et al. Preventing depression among early adolescents in the primary care setting: a randomized controlled study of the Penn Resiliency Program. *J Abnorm Child Psychol* 2006;34:203–19. doi:10.1007/s10802-005-9014-7

10. Seligman MEP, Schulman P, Tryon AM. Group prevention of depression and anxiety symptoms. *Behav Res Ther* 2007;45:1111–26. doi:10.1016/j.brat.2006.09.010

11. Stice E, Burton E, Bearman SK, et al. Randomized trial of a brief depression prevention program: an elusive search for a psychosocial placebo control condition. *Behav Res Ther* 2007;45:863–76. doi:10.1016/j.brat.2006.08.008

12. Horowitz JL, Garber J. The prevention of depressive symptoms in children and adolescents: A meta-analytic review. *J Consult Clin Psychol* 2006;74:401–15. doi:10.1037/0022-006X.74.3.401

13. Merry S, McDowell H, Hetrick S, et al. Psychological and/or educational interventions for the prevention of depression in children and adolescents. *Cochrane Database Syst Rev* 2004;:CD003380. doi:10.1002/14651858.CD003380.pub2

14. Roberts CM, Kane R, Bishop B, et al. The prevention of anxiety and depression in children from disadvantaged schools. *Behav Res Ther* 2010;48:68–73. doi:10.1016/j.brat.2009.09.002

15. Sawyer MG, Pfeiffer S, Spence SH, et al. School-based prevention of depression: a randomised controlled study of the beyondblue schools research initiative. *J Child Psychol Psychiatry* 2010;51:199–209. doi:10.1111/j.1469-7610.2009.02136.x

16. Klein DN, Glenn CR, Kosty DB, *et al.* Predictors of first lifetime onset of major depressive disorder in young adulthood. *J Abnorm Psychol* 2013;122:1–6. doi:10.1037/a0029567

17. Chan A-W, Tetzlaff JM, Altman DG, Laupacis A, Gøtzsche PC, Krleža-Jerić K, Hróbjartsson A, Mann H, Dickersin K, Berlin J, Doré C, Parulekar W, Summerskill W, Groves T, Schulz K, Sox H, Rockhold FW, Rennie D, Moher D. SPIRIT 2013 Statement: Defining standard protocol items for clinical trials. Ann Intern Med. 2013;158(3):200-207

18. Ivarsson T, Svalander P, Litlere O. The Children's Depression Inventory (CDI) as measure of depression in Swedish adolescents. A normative study. *Nordic Journal of Psychiatry*, 60 (3) (2006), pp. 220-226

19. Sheehan, D.V.; Sheehan, K.H.; Shyte, R.D.; Janavs, J.; Bannon, Y.; Rogers, J.E.; Milo, K.M.; Stock, S.L.; Wilkinson, B. Reliability and validity of the Mini International Neurpsychiatric Interview for Children and Adolescents (MINI-KID). *J. Clin. Psychiatry* 2010, 71, 313–326.

20. Chorpita, B. F., Moffitt, C., & Gray, J. (2005). Psychometric properties of the Revised Child Anxiety and Depression Scale in a clinical sample. *Behaviour Research and Therapy*, 43, 309-322.

21. American Psychiatric Association. (2013). Diagnostic and statistical manual of mental disorders (5th ed.). Washington, DC: Author.

22. Shaffer, D., Gould, M.S., Brasic, J., Ambrosini, P., Fisher, P., Bird, H., & Aluwahlia, S. (1983). A Children's Global Assessment Scale (CGAS). *Archives of General Psychiatry*, 40, 1228-1231.

23. Mundt, J.C., Marks, I.M., Shear K.M., Greist J., (2002). Impairment in functioning. The work and social adjustment scale: a simple measure of impairment of functioning. *The British Journal of Psychiatry*, 180, 461-464.

24. Keyes, C. L. M. (2009). Atlanta: Brief description of the mental health continuum short form (MHC-SF). Available: https://www.aacu.org/sites/default/files/MHC-SFEnglish.pdf. [On–line, retrieved 2020-03-30]

25. Keyes CLM, Wissing M, Potgieter JP, *et al.* Evaluation of the mental health continuum-short form (MHC-SF) in setswana-speaking South Africans. *Clin Psychol Psychother* 2008;15:181–92. doi:10.1002/cpp.572

For peer review only

26. Keyes CLM. The Subjective Well-Being of America's Youth: Toward a Comprehensive Assessment. *Adolescent & Family Health* 2006;4:3–11.

27. Lamers SMA, Glas CAW, Westerhof GJ, *et al.* Longitudinal evaluation of the mental health continuum-short form (MHC-SF): Measurement invariance across demographics, physical illness, and mental illness. *Eur J Psychol Assess* 2012;28:290–6. doi:10.1027/1015-5759/a000109

For peer review only

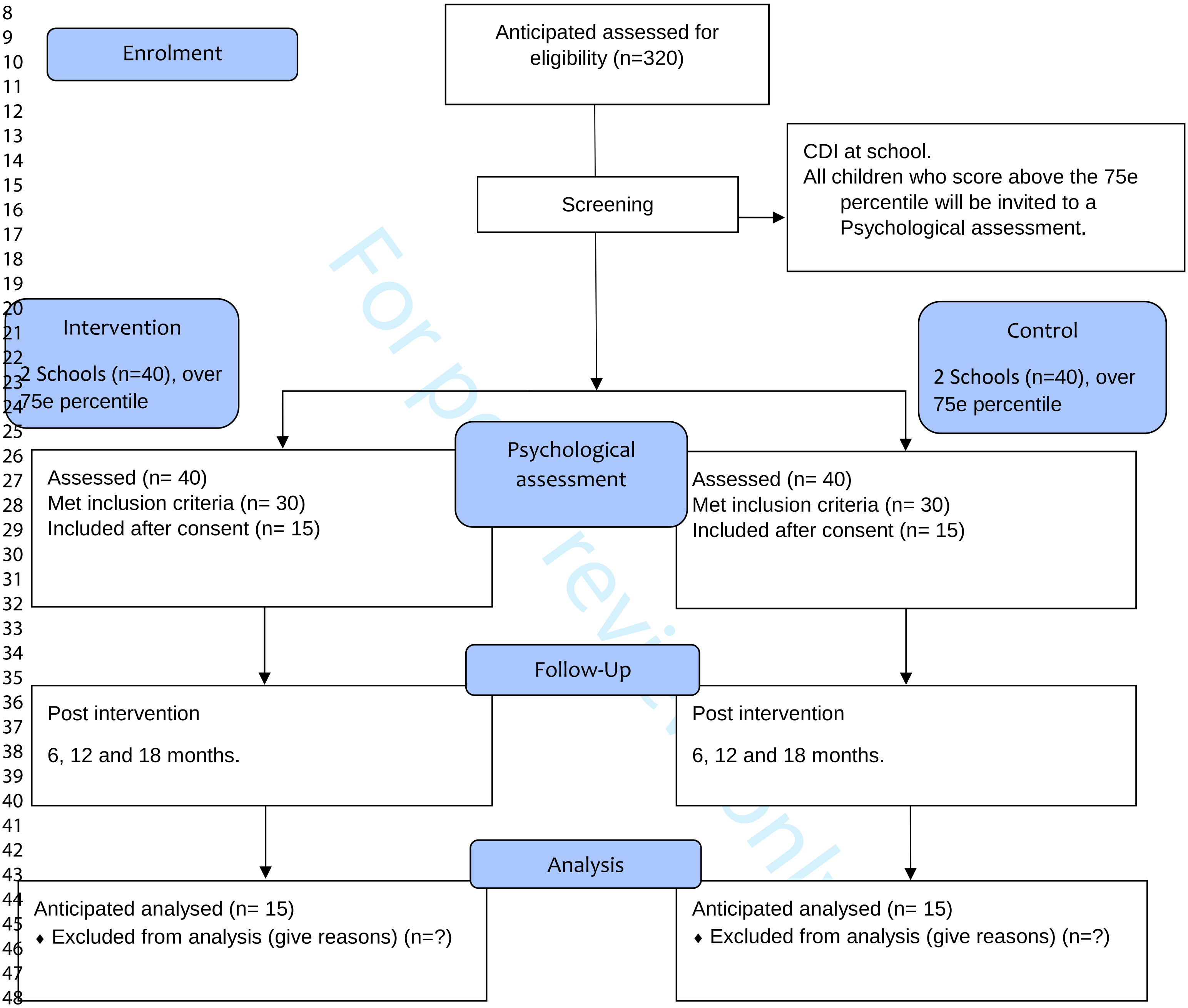
Enrolment
Anticipated assessed for eligibility (n=320)
Screening
CDI at school.
All children who score above the 75e percentile will be invited to a Psychological assessment.
Intervention
2 Schools (n=40), over 75e percentile
Control
2 Schools (n=40), over 75e percentile
Psychological assessment
Assessed (n= 40)
Met inclusion criteria (n= 30)
Included after consent (n= 15)
Assessed (n= 40)
Met inclusion criteria (n= 30)
Included after consent (n= 15)
Follow-Up
Post intervention
6, 12 and 18 months.
Post intervention
6, 12 and 18 months.
Analysis
Anticipated analysed (n= 15)
♦ Excluded from analysis (give reasons) (n=?)
Anticipated analysed (n= 15)
♦ Excluded from analysis (give reasons) (n=?)

Reporting checklist for protocol of a clinical trial.

Based on the SPIRIT guidelines.

Instructions to authors

Complete this checklist by entering the page numbers from your manuscript where readers will find each of the items listed below.

Your article may not currently address all the items on the checklist. Please modify your text to include the missing information. If you are certain that an item does not apply, please write "n/a" and provide a short explanation.

Upload your completed checklist as an extra file when you submit to a journal.

In your methods section, say that you used the SPIRITreporting guidelines, and cite them as:

Chan A-W, Tetzlaff JM, Altman DG, Laupacis A, Gøtzsche PC, Krleža-Jerić K, Hróbjartsson A, Mann H, Dickersin K, Berlin J, Doré C, Parulekar W, Summerskill W, Groves T, Schulz K, Sox H, Rockhold FW, Rennie D, Moher D. SPIRIT 2013 Statement: Defining standard protocol items for clinical trials. Ann Intern Med. 2013;158(3):200-207

		Reporting Item	Page Number
**Administrative information**			
Title	#1	Descriptive title identifying the study design, population, interventions, and, if applicable, trial acronym	1

For peer review only

Trial registration	#2a	Trial identifier and registry name. If not yet registered, name of intended registry	1
Trial registration: data set	#2b	All items from the World Health Organization Trial Registration Data Set	n/a
Protocol version	#3	Date and version identifier	n/a
Funding	#4	Sources and types of financial, material, and other support	11
Roles and responsibilities: contributorship	#5a	Names, affiliations, and roles of protocol contributors	1
Roles and responsibilities: sponsor contact information	#5b	Name and contact information for the trial sponsor	n/a
Roles and responsibilities: sponsor and funder	#5c	Role of study sponsor and funders, if any, in study design; collection, management, analysis, and interpretation of data; writing of the report; and the decision to submit the report for publication, including whether they will have ultimate authority over any of these activities	n/a
Roles and responsibilities: committees	#5d	Composition, roles, and responsibilities of the coordinating centre, steering committee, endpoint adjudication committee, data management team, and	n/a

For peer review only

		other individuals or groups overseeing the trial, if applicable (see Item 21a for data monitoring committee)	
**Introduction**			
Background and rationale	#6a	Description of research question and justification for undertaking the trial, including summary of relevant studies (published and unpublished) examining benefits and harms for each intervention	2
2Background and rationale: choice of comparators	#6b	Explanation for choice of comparators	2
Objectives	#7	Specific objectives or hypotheses	2
Trial design	#8	Description of trial design including type of trial (eg, parallel group, crossover, factorial, single group), allocation ratio, and framework (eg, superiority, equivalence, non-inferiority, exploratory)	3
**Methods: Participants, interventions, and outcomes**			
Study setting	#9	Description of study settings (eg, community clinic, academic hospital) and list of countries where data will be collected. Reference to where list of study sites can be obtained	3

For peer review only

Eligibility criteria	#10	Inclusion and exclusion criteria for participants. If applicable, eligibility criteria for study centres and individuals who will perform the interventions (eg, surgeons, psychotherapists)	3
Interventions: description	#11a	Interventions for each group with sufficient detail to allow replication, including how and when they will be administered	7
Interventions: modifications	#11b	Criteria for discontinuing or modifying allocated interventions for a given trial participant (eg, drug dose change in response to harms, participant request, or improving / worsening disease)	n/a
Interventions: adherance	#11c	Strategies to improve adherence to intervention protocols, and any procedures for monitoring adherence (eg, drug tablet return; laboratory tests)	n/a
Interventions: concomitant care	#11d	Relevant concomitant care and interventions that are permitted or prohibited during the trial	n/a
Outcomes	#12	Primary, secondary, and other outcomes, including the specific measurement variable (eg, systolic blood pressure), analysis metric (eg, change from baseline, final value, time to event), method of aggregation (eg, median, proportion), and time point for each outcome. Explanation of the clinical relevance of chosen efficacy and harm outcomes is strongly recommended	5

For peer review only

Participant timeline	#13	Time schedule of enrolment, interventions (including any run-ins and washouts), assessments, and visits for participants. A schematic diagram is highly recommended (see Figure)	10
Sample size	#14	Estimated number of participants needed to achieve study objectives and how it was determined, including clinical and statistical assumptions supporting any sample size calculations	7
Recruitment	#15	Strategies for achieving adequate participant enrolment to reach target sample size	4
**Methods: Assignment of interventions (for controlled trials)**			
Allocation: sequence generation	#16a	Method of generating the allocation sequence (eg, computer-generated random numbers), and list of any factors for stratification. To reduce predictability of a random sequence, details of any planned restriction (eg, blocking) should be provided in a separate document that is unavailable to those who enrol participants or assign interventions	n/a
Allocation concealment mechanism	#16b	Mechanism of implementing the allocation sequence (eg, central telephone; sequentially numbered, opaque,	n/a

For peer review only

		sealed envelopes), describing any steps to conceal the sequence until interventions are assigned	
Allocation: implementation	#16c	Who will generate the allocation sequence, who will enrol participants, and who will assign participants to interventions	n/a
Blinding (masking)	#17a	Who will be blinded after assignment to interventions (eg, trial participants, care providers, outcome assessors, data analysts), and how	n/a
Blinding (masking): emergency unblinding	#17b	If blinded, circumstances under which unblinding is permissible, and procedure for revealing a participant's allocated intervention during the trial	n/a
**Methods: Data collection, management, and analysis**			
Data collection plan	#18a	Plans for assessment and collection of outcome, baseline, and other trial data, including any related processes to promote data quality (eg, duplicate measurements, training of assessors) and a description of study instruments (eg, questionnaires, laboratory tests) along with their reliability and validity, if known. Reference to where data collection forms can be found, if not in the protocol	10

For peer review only

Data collection plan: retention	#18b	Plans to promote participant retention and complete follow-up, including list of any outcome data to be collected for participants who discontinue or deviate from intervention protocols	n/a
Data management	#19	Plans for data entry, coding, security, and storage, including any related processes to promote data quality (eg, double data entry; range checks for data values). Reference to where details of data management procedures can be found, if not in the protocol	7
Statistics: outcomes	#20a	Statistical methods for analysing primary and secondary outcomes. Reference to where other details of the statistical analysis plan can be found, if not in the protocol	7
Statistics: additional analyses	#20b	Methods for any additional analyses (eg, subgroup and adjusted analyses)	n/a
Statistics: analysis population and missing data	#20c	Definition of analysis population relating to protocol non-adherence (eg, as randomised analysis), and any statistical methods to handle missing data (eg, multiple imputation)	n/a

Methods: Monitoring

Data monitoring: formal committee	#21a	Composition of data monitoring committee (DMC); summary of its role and reporting structure; statement of whether it is independent from the sponsor and competing interests; and reference to where further	n/a

For peer review only

		details about its charter can be found, if not in the protocol. Alternatively, an explanation of why a DMC is not needed	
Data monitoring: interim analysis	#21b	Description of any interim analyses and stopping guidelines, including who will have access to these interim results and make the final decision to terminate the trial	n/a
Harms	#22	Plans for collecting, assessing, reporting, and managing solicited and spontaneously reported adverse events and other unintended effects of trial interventions or trial conduct	8
Auditing	#23	Frequency and procedures for auditing trial conduct, if any, and whether the process will be independent from investigators and the sponsor	n/a
**Ethics and dissemination**			
Research ethics approval	#24	Plans for seeking research ethics committee / institutional review board (REC / IRB) approval	8
Protocol amendments	#25	Plans for communicating important protocol modifications (eg, changes to eligibility criteria, outcomes, analyses) to relevant parties (eg, investigators, REC / IRBs, trial participants, trial registries, journals, regulators)	n/a

For peer review only

Consent or assent	#26a	Who will obtain informed consent or assent from potential trial participants or authorised surrogates, and how (see Item 32)	8
Consent or assent: ancillary studies	#26b	Additional consent provisions for collection and use of participant data and biological specimens in ancillary studies, if applicable	n/a
Confidentiality	#27	How personal information about potential and enrolled participants will be collected, shared, and maintained in order to protect confidentiality before, during, and after the trial	n/a
Declaration of interests	#28	Financial and other competing interests for principal investigators for the overall trial and each study site	11
Data access	#29	Statement of who will have access to the final trial dataset, and disclosure of contractual agreements that limit such access for investigators	7
Ancillary and post trial care	#30	Provisions, if any, for ancillary and post-trial care, and for compensation to those who suffer harm from trial participation	
Dissemination policy: trial results	#31a	Plans for investigators and sponsor to communicate trial results to participants, healthcare professionals, the public, and other relevant groups (eg, via publication, reporting in results databases, or other data sharing arrangements), including any publication restrictions	n/a

For peer review only

Dissemination policy: authorship	#31b	Authorship eligibility guidelines and any intended use of professional writers	n/a
Dissemination policy: reproducible research	#31c	Plans, if any, for granting public access to the full protocol, participant-level dataset, and statistical code	n/a

Appendices

Informed consent materials	#32	Model consent form and other related documentation given to participants and authorised surrogates	n/a
Biological specimens	#33	Plans for collection, laboratory evaluation, and storage of biological specimens for genetic or molecular analysis in the current trial and for future use in ancillary studies, if applicable	n/a

None The SPIRIT checklist is distributed under the terms of the Creative Commons Attribution License CC-BY-ND 3.0. This checklist can be completed online using https://www.goodreports.org/, a tool made by the EQUATOR Network in collaboration with Penelope.ai

For peer review only

BMJ Open

Is the “Thoughts and Health” programme feasible in the context of Swedish schools, a quasi-experimental controlled trial - Study Protocol

Journal:	*BMJ Open*
Manuscript ID	bmjopen-2020-040374.R1
Article Type:	Protocol
Date Submitted by the Author:	22-Oct-2020
Complete List of Authors:	Wikberg, Carl; University of Gothenburg Institute of Medicine, School of Public Health and Community Medicine<br>Augustsson, Pia; University of Gothenburg Department of Public Health and Community Medicine, ;<br>Sveinsdottir, Gudny; Vastra Gotaland Primary Health Care, Närhälsan Research and Development Primary Health Care, Region Västra Götaland, Gothenburg, Sweden<br>Craighead, Edward; Emory University, Department of Psychology, Psychiatry & Behavioral Sciences<br>Arnarson, Erikur<br>Marteinsdottir, Ina; University of Kalmar, Department of Medicine and Optometry<br>Lilja, Josefine L.; University of Gothenburg Department of Psychology, Department of Psychology, University of Gothenburg, Gothenburg, Sweden
**Primary Subject Heading**:	Mental health
Secondary Subject Heading:	General practice / Family practice, Public health, Mental health
Keywords:	Child & adolescent psychiatry < PSYCHIATRY, PUBLIC HEALTH, Depression & mood disorders < PSYCHIATRY

SCHOLARONE™
Manuscripts

I, the Submitting Author has the right to grant and does grant on behalf of all authors of the Work (as defined in the below author licence), an exclusive licence and/or a non-exclusive licence for contributions from authors who are: i) UK Crown employees; ii) where BMJ has agreed a CC-BY licence shall apply, and/or iii) in accordance with the terms applicable for US Federal Government officers or employees acting as part of their official duties; on a worldwide, perpetual, irrevocable, royalty-free basis to BMJ Publishing Group Ltd ("BMJ") its licensees and where the relevant Journal is co-owned by BMJ to the co-owners of the Journal, to publish the Work in this journal and any other BMJ products and to exploit all rights, as set out in our licence.

The Submitting Author accepts and understands that any supply made under these terms is made by BMJ to the Submitting Author unless you are acting as an employee on behalf of your employer or a postgraduate student of an affiliated institution which is paying any applicable article publishing charge ("APC") for Open Access articles. Where the Submitting Author wishes to make the Work available on an Open Access basis (and intends to pay the relevant APC), the terms of reuse of such Open Access shall be governed by a Creative Commons licence – details of these licences and which Creative Commons licence will apply to this Work are set out in our licence referred to above.

Other than as permitted in any relevant BMJ Author's Self Archiving Policies, I confirm this Work has not been accepted for publication elsewhere, is not being considered for publication elsewhere and does not duplicate material already published. I confirm all authors consent to publication of this Work and authorise the granting of this licence.

For peer review only

Is the "Thoughts and Health" programme feasible in the context of Swedish schools, a quasi-experimental controlled trial - Study Protocol

Carl Wikberg[1,2], Pia Augustsson[1,2], Gudny Sveinsdottir[2] , W Edward Craighead[3], Eirikur Örn Arnarson[4], Ina Marteinsdottir[5], Josefine L Lilja[2,6].

Affiliation:

1 School of Public Health and Community Medicine, Institute of Medicine, Sahlgrenska Academy, University of Gothenburg, Gothenburg, Sweden.

2 Närhälsan Research and Development Primary Health Care, Region Västra Götaland, Gothenburg, Sweden.

3 Department of Psychiatry and Behavioral Sciences and the Department of Psychology, Emory University, 12 Executive Park Drive NE, Suite 200, Atlanta, GA 30329, USA.

4 Department of Psychiatry, Landspítali-University Hospital, Reykjavík, Iceland.

5 Department of Clinical and Experimental Medicine, Center for Social and Affective Neuroscience, Linköping University, 581 85, Linköping, Sweden.

6 Department of Psychology, University of Gothenburg, Gothenburg, Sweden.

Corresponding author

Carl Wikberg – carl.wikberg@allmed.gu.se

Box 428, 405 30 Gothenburg

+46703821660

Keywords: Depression, Adolescence, Prevention, Feasibility

Word count: 4862

For peer review only

Abstract

Introduction; Clinical depression is a substantial problem among adolescents, increasing significantly at about age 15. It causes impairment in social, academic, and familial relationships as well as ongoing cognitive and emotional difficulties for the individual. A study in Iceland demonstrated that a cognitive-behavioural, developmentally based intervention programme, “Thoughts and Health”, prevented initial episodes of depression and/or dysthymia (MDD/DYS) in adolescents for up to 12 months following completion of the programme. We would like to test the feasibility of implementing the Icelandic method in a Swedish context and to evaluate the long-term effects of such a programme.

Methods and analysis; A quasi-experimental controlled design combined with qualitative and quantitative methods will be used to address the research questions.

In this study 617 children aged ~14 will be screened for depression and those “at risk” for development of clinical depression will be offered a 12 week course “Thoughts and Health”. This course aims to prevent first depression in adolescents. A comparable group of children will function as control.

Depending on type of variable, baseline comparisons of the two groups of relevant initial measures will be evaluated with t-tests or chi-square analyses. The effects of the programme on the development of clinical levels of depression will be evaluated for the 6-, 12-, and 18-month data. Index parental depression at baseline will be tested as a moderator in the evaluation of the prevention programme effects.

Ethics and dissemination.

This study is approved by the Swedish Ethical Review Board (reference number 2019-03347) in Gothenburg.

We plan to disseminate the knowledge gained from this study by publishing our results in peer-reviewed scientific journals and other scholarly outlets.

Registration details: NCT04128644 Clinical trials.

Strengths and limitations of this study

- Quasi-experimental controlled design will provide information about whether the intervention prevents depressive episodes in adolescence in a Swedish sample.
- Qualitative data will shed light on how the intervention was perceived by the adolescents and how the personnel experienced the implementation process.
- Quantitative data will provide information regarding adolescents’ mental health and social functioning.
- Follow-up assessments will provide information regarding the intervention’s long-term effects on school performance and mental health.
- The study lacks a randomised controlled design and has few participants, which limit its generalisability.

For peer review only

Introduction

Clinical depression is a substantial problem among adolescents, increasing significantly at about age 15. It causes impairment in social, academic, and familial relationships as well as ongoing cognitive and emotional difficulties for the individual [1, 2].

A study in Iceland demonstrated that a cognitive-behavioural, developmentally based intervention programme called "Thoughts and Health" prevented initial episodes of depression and/or dysthymia (MDD/DYS) among Icelandic adolescents for up to 12 months following completion of the programme [3,4]. Other studies have demonstrated that similar programmes can be employed to prevent depression among teens who have never been depressed and/or who previously have been depressed [5–8]. The "Thought and Health" programme utilises principles of both behavioral (e.g., relaxation, behavioral activation (assertion and social approach)) and cognitive (e.g., identification and modification of irrational thoughts, thinking errors, and self-beliefs) interventions. The programme procedures were specifically adapted to the appropriate developmental level of the youth participants, with developmental level defined as Stage 5 (Identity vs Confusion) of Erikson's conceptual model of human psychosocial development[9]. Specifically, Stage 5 encompasses adolescence during which individuals *develop a sense of self* within the context of emerging independence from the family and expansion of interpersonal/social relationships.

However, despite the positive impact of such programmes, preventive effects have not been consistently obtained [10–12]. Reviews have concluded that universal prevention programmes (programmes applied to the general population) have not been effective in preventing depression (see [13, 14]), and excellently conducted studies in Australia have provided data consistent with that conclusion [15, 16]. In general, only targeted programmes (*selective* programmes–for populations exposed to risk factors or *indicated* programmes–for populations who are experiencing some symptoms) have shown significant short-term effects in preventing depression (e.g., [1, 5–7]).

Most programmes aimed at preventing depression have included both youth who have suffered a prior depression and those who have never been depressed. The study of prevention of relapse or recurrence in prevention programmes for those who have already suffered a major depressive disorder (MDD) is clearly useful, but earlier studies have not distinguished between prevention of the first episode of MDD and the prevention of relapse/recurrence of MDD. However, because of the recurrent nature of the disorder and the differing causal factors and probability patterns of first versus subsequent episodes [17] and in order to prevent the deleterious effects of an episode of MDD in youth, it is especially important to study the prevention of the first or initial episode among never-previously-depressed subjects.

Since clinical depression among adolescents increases significantly at about age 15, and Swedish children finish their compulsory school (junior high school) at that age, being at risk for depression can be a reason for failing their final examinations, thereby limiting their chances for further academic studies.

We would like to test the feasibility of implementing the Icelandic method in a Swedish context and to evaluate the long-term effects of such a programme. We would like to test the method with a quasi-experimental controlled trial, and evaluate the outcomes after 6, 12 and 18 months. The control group will be assessment-only adolescents, they receive ordinary "Student health", they participate in all study assessments, but they are not offered the intervention, "Thoughts and health"

This is, to our knowledge, the first study in Sweden that employs a longitudinal design to evaluate the effects of a school-based programme aimed at preventing depression in adolescents.

For peer review only

This study will be implemented together and within the municipalities of Tjörn and Orust, two islands off the west coast of Sweden, with each island only having two middle schools. Thus, this study is built on a close collaboration between academia, primary care, and the community.

We are hopeful that this programme will be transferrable to a Swedish context and that it will be possible to further develop and enhance the method. Iceland and Sweden are admittedly similar in many ways, but the school system and cultural context differs to some extent.

We hypothesise that programme implementation will reduce the risk of developing depressive symptoms among participating students compared to their control counterparts, and we will examine this in participating adolescents up to 18 months after programme completion. We also hypothesise that adolescents in the intervention group who participate in the programme will finish school with complete grades to a higher extent (more students complete their schooling) than the control group.

Aim

This study aims to test the feasibility of implementing an Icelandic cognitive-behavioural programme designed to prevent depression, "Thoughts and Health" [3,4], in a Swedish school setting.

The perspectives of both the participating adolescents and the primary care professionals who lead and support the programme in the schools will be taken into account when evaluating the programme's feasibility.

The study also aims to evaluate the number of students who finish junior high school (ninth grade, ~15 years old) with complete grades. In this study, the outcomes concerning complete grades of the students participating in the intervention programme will be compared to the complete grades of those in the control group.

Our primary research questions are:

Is the "Thoughts and Health" programme feasible in the context of Swedish schools, from the perspective of the students participating in the programme?

Is the "Thoughts and Health" programme feasible in the context of Swedish schools, from the perspective of the primary care professionals leading and supporting the programme implementation?

Our secondary research questions are:

Does participation in the "Thoughts and Health" programme affect the number of students developing depressive symptoms?

Will the participation in the course "Thoughts and Health" increase the participants' chances of completing compulsory school with high school eligibility?

Method

Study design

A quasi-experimental controlled design combined with qualitative and quantitative methods will be used to address the research questions. NCT04128644 , Clinicaltrials.com

We have used the SPIRIT reporting guidelines [18]

Participants

An invitation regarding participation in the study to high schools in the area resulted in four junior high schools from the municipality of Tjörn (Häggvall and Bleket, intervention arm) and Orust (Henån och Ängås, control arm) who will participate in the study. The allocation of the schools was

For peer review only

chosen on the basis of their rural location and socioeconomic similarity, willingness to participate, and available staff. Since all schools had shown interest in participating in this study, the assignment of intervention and control were determined on available recourses; therefore, Tjörn were chosen for the intervention programme at this stage. Two intervention courses are planned (I and II), with course I beginning at the start of the fall semester (~1st September) 2019 and course II beginning at the start of the fall semester (~1st September) 2020.

In total, approximately 320 eighth grade Swedish adolescents aged 14 years will participate in a pre-intervention screening process per course. In the pre-intervention screening process, all students will complete the Children's Depression Inventory (CDI, described below) [19] assessment instrument via an online questionnaire tool. This will be part of the existing "Student Health" ordinary assignments that include health and mental health status checkups as per the Swedish School Law (Skollagen 2010:800).

We are aware of the small number of students that will be part of the collected data at this early stage, and we are planning a full scale randomised trial once we have completed this first study; hence, there were no power calculations of sample size at this stage.

All students who score at or above the 75th percentile on the CDI screening will be qualified initially for possible inclusion in the study (expected n=80 total per course). They will receive an invitation together with their parents to attend a psychological assessment interview (the Mini International Neuropsychiatric Interview for Children and Adolescents ((MINI-KID), described below) [20]. At the interview, they will be given the opportunity to discuss their CDI result and if needed, to be channeled to the right level of care. (Example: If a student scored very high on CDI, the psychologist will need to decide whether there is any underlying diagnosis that requires treatment or if the student has any other condition that also requires immediate attention and treatment.) If a student is diagnosed with depression at this phase, he or she will no longer be eligible to participate in the study and will be directed to appropriate mental health care resources in the community. Prior to attending this psychological assessment, students and their parents will separately fill out the Revised Children's Anxiety and Depression Scale (RCADS) [21] assessment instrument.

Based on the MINI-KID results, students who meet criteria for having any current or past major depression, dysthymia, bipolar disorder I or II, cyclothymia, anorexia, bulimia, psychotic disorder, alcohol or substance dependence, attention deficit hyperactivity disorder (ADHD), oppositional defiant disorder (ODD), or conduct disorder will be excluded. All other students thus fulfill inclusion criteria. All students, both in the intervention and control arms, will be invited to participate in the study via an appropriately-worded letter (expected n=40 total per study arm). See table 1 for inclusion and exclusion criteria.

Thus, about 20 students per course will be offered to participate in the intervention programme. These students will be approached by the programme staff and offered the opportunity to participate in the baseline clinical interview and assessment, the intervention programme, and post-intervention follow-up assessments at 1, 6, 12, and 18 months after programme completion (figure 1).

Participants in the intervention and control arm will be offered tickets to the cinema as a gesture of gratitude for their participation in each of the follow-up assessments.

Measures and assessments

Measures and assessments are described below. Who and the time point for each measure or assessment will show in table 2. All psychometric measures are administrated by esMaker (htpps://entergate.se/products/esmaker/). Course surveys are handed out (hard copys). If participant frequency is shown to be low, we will remind those who have not completed the measures by email.

For peer review only

Outcomes

CDI, ordinal variables (collected during pre-intervention phase, at course start, and at 6-, 12-, and 18-months follow-up). The CDI is a 27-item self-reported, symptom-oriented scale that rates the severity of depression during the past month. It is designed for children/adolescents, ages 7-17 years. Each of the 27 items comprises 3 sentences used by the student to rate the severity of the symptoms. This inventory has previously shown excellent internal consistency (alpha coefficients of .83 to .94). The present study utilises the total score of the Swedish version CDI total score [19]. The CDI will be used both in screening and in follow ups with participating children.

The Revised Children's Anxiety and Depression Scale (RCADS), ordinal variables (collected during pre-intervention phase and at 6-, 12-, and 18-months follow-up). RCADS is a widely used questionnaire designed to assess clinical syndromes of anxiety as well as depression, building on the DSM-IV criteria. The RCADS provides two total scores, and 6 subscales: Separation Anxiety Disorder (SAD), Social Phobia (SoP), Obsessive Compulsive Disorder (OCD), Panic Disorder (PD), Generalised Anxiety Disorder (GAD), and Major Depressive Disorder (MDD). The internal consistency of the RCADS subscales is high, with Cronbach alphas ranging from 0.78 to 0.88. There are two versions of RCADS; one for adolescents (RCADS) and one for parents (RCADS-P).

MINI-KID, categorical variables (collected during pre-intervention phase and at 6-, 12-, and 18-months follow-up). The MINI-KID is a structured interview for children and young people aged 4-17 years old[20]. It is based on diagnoses using the Diagnostic and Statistical Manual of Mental Disorders (DSM-5) [22] and the tenth edition of International Statistical Classification of Diseases and Related Health Problems (ICD-10). Both parents and youths will participate in the interview, but the interview can also be conducted with only young people. The interview aims to ensure diagnosis and inclusion/exclusion criteria.

Demographic information and somatic health, categorical variables (collected at course start and at 6-, 12-, and 18-months follow-up). The baseline questionnaire will include items regarding demographics, physical activity, and drug use, and some questions about mobile and internet usage

Children's Global Assessment Scale (CGAS)[23], ordinal variables (collected during pre-intervention phase). The CGAS will be used to measure global functioning. The assessment will be made by the person who conducts the MINI-KID interview. The CGAS estimate is made on a scale of 1-100, where a higher score indicates a better functioning of life / higher functional level. This will be completed for participating children.

Education Work and Social Adjustment Scale (EWSAS) [24], ordinal variables (collected at course start, course middle (6 weeks after course start), and at 6-, 12-, and 18-months follow-up). The EWSAS measures general level of function. It is an adaptation of the Work and Social adjustment Scale (WSAS) and measures the level of function in five areas: relationships, family, leisure time, social activities, and school and everyday activities. This will be completed by participating children.

Mental Health Continuum – Short Form (MHC – SF), ordinal variables (collected at course start, course middle (6 weeks after course start), and at 6-, 12-, and 18-months follow-up). The MHC-SF is an increasingly used questionnaire intending to measure three components of well-being: emotional, social, and psychological. It has shown excellent internal consistency (>.80) and discriminant validity in adolescents (ages 12-18 years) and adults in the U.S., in the Netherlands, and in South Africa [25-28]. MHC-SF consists of 14 items asking about the frequency of well-being experiences during the last month. There are three items concerning emotional well-being such as feelings of being happy, interested in life and satisfied, five items concerning social well-being, reference, actualisation, contribution, coherence and integration, and six items concerning psychological well-being capturing purpose in life, environmental mastery, autonomy, personal growth, positive relations and self-acceptance. Answers are given on a 6-point Likert scale from 0 = never, 1 = once or twice, 2 = about

For peer review only

once a week, 3 = about 2 or 3 times a week, 4 = almost every day, 5 = every day. Cronbach's alpha was 0.92 for emotional well-being, 0.88 for social well-being and 0.92 for psychological well-being. Scores on each well-being component are summed and categorised as "flourishing", "moderately healthy" or "languishing" according to official MHC-SF guidelines. The MHC-SF has been useful in improving the scientific understanding of the risk of future mental illness (major depressive episode, GAD, panic attacks, risk of premature mortality, healthcare utilisation, missed days of work, suicidality, and self-reported academic impairment among college students). The MHC-SF will be completed by participating children.

Parental Depression, binary variable (collected during pre-intervention phase). At the time of the baseline interview, adolescents will be asked if their parents previously have suffered from or currently suffer from depression. Similarly, parents will be asked if they have previously suffered from or currently suffer from depression. These data are collected because there are known associations between depression in parents and the health of their children (6). We would like to know if any of the parents have had or currently have a depressive episode as that may be related to the depressive outcomes in their children. Both adolescents and parents data will be analysed separately. This will be useful for further analysis regarding long term follow ups. As an example we will be able to see that both intervention group and control group parents are similar regarding possible prior or current state of depression.

Course surveys, ordinal variables (collected after each course session). After each course session (total of 12 sessions per course), the students and the primary care professionals leading and supporting the course will directly provide their feedback about the course session by answering a short survey. Different surveys will be provided to students and the professionals, respectively. These surveys will assess feasibility, satisfaction, and overall impression of the session.

Course attendance and dropouts, binary variables (collected during each course session). Attendance and dropouts will be recorded at each course meeting, as a measure of the feasibility and acceptance of the programme.

Focus groups and interviews, qualitative analysis (collected within 1 month after course end and at 18-months follow-up). All participants (students and professionals) will be invited to take part in focus group discussions or individual interviews after the completion of the full course. These discussions will be held separately, with students in one or two groups and the professionals in another, or as individual interviews. Focus groups will be held with the students, and individual interviews with professionals.

Final junior high grades, categorical variables (collected at approximately 18 months after course end). Students' final grades in junior high school (that is, in ninth grade, or 18 months after their inclusion in the programmes) will be collected to be able to evaluate the number of students who finish school with complete grades. The information will be provided by the school administration upon consent from the students.

All psychological assessments will be conducted by a trained psychologist from the Primary Care Centre, and the focus groups and interviews will be conducted by the authors CW (PhD and Specialist nurse) and PA (PhD student)

Data management

A research journal follows the person during the programme and follow-up period. The research journal will be kept at the primary care R&D unit, Kungsgatan, in Gothenburg, where data from the research journal are entered. Data in the database at the research unit will only be entered with code numbers. Only the research leaders will then have access to the code key, which is stored in locked (in safes) at the research unit. The research journals are kept anonymous at the research unit during the

For peer review only

project evaluation phase. The composition of the research group may change during the course of the project, but only authorised persons who are part of the research group will have access to data. The usual privacy is maintained.

Intervention: Prevention Programme

The prevention programme will be based on the Icelandic “Thought and Feelings” programme [1] and will include 12 group sessions of 90-minutes duration, delivered twice per week for two weeks and then once a week for eight weeks in the school setting by “Student Health” school psychologists. School nurses will also attend each session as support for the students. The programme uses two intervention manuals: a group leader manual and a student manual. Both of these manuals have been translated first from Icelandic to English, and then from the English version into Swedish, and finally corrected for language in the Swedish version. After translation, the manuals were additionally reviewed by several eighth-grade students for cultural acceptability (e.g., word choice) and adapted as per their feedback. The course material is based on cognitive behaviour theory, and the course will be held as a group activity. The course was originally developed as a group activity, and we will test this method in our study, as part of the evaluation of whether the method is suitable for Swedish adolescents of today. The course contains group sessions with a specific subject, after which they will describe their state of health and feelings in a diary and receive some home assignments to be completed between sessions. All sessions start with a light refreshment and a résumé (Summary) of the last session. Session #1 is an introduction. Session #2 is about “the spiral”, that is, how negative or positive thoughts and actions affect subsequent actions and feelings. Session #3 is about relaxation. Session #4 will discuss the concept of a “baseline”. The remaining sessions deal with the following topics: setting targets (#5), feelings (#6), negative thoughts (#7), irrational thoughts (#8), self-perception (#9), communication (#10), stop and reconsideration (#11) and consummation (#12, final session).

Statistical Analysis

Depending on type of variable, baseline comparisons of the two groups of relevant initial measures will be evaluated with t-tests or chi-square analyses. The effects of the programme on the development of depressive symptoms will be evaluated for the 6-, 12-, and 18-month data using the Cox proportional hazards model to estimate survival curves and rates for different thresholds of combined scores of depressive symptoms. Index parental depression at baseline will be tested as a moderator in the evaluation of the prevention programme effects. Even though the sample in this part of the project is rather small, we will stratify all results by different variables in the baseline questionnaire including gender, socioeconomic status, and mobile/internet use, among others. With regard to the small sample size in this early stage of the study, drop outs or non completion of outcome measures will be analysed with intent-to-treat.

The qualitative data collected through interviews and focus group discussions will not require any statistical analysis, but they will be analysed using a qualitative analytical methods named thematic analysis.

Ethics and Dissemination

This study is approved by the Swedish Ethical Review Board (reference number 2019-03347) in Gothenburg. Since participants are adolescents <15 years, consent from both parents (all parents if eligible) and the adolescents themselves will be obtained, as required by applicable laws and regulations.

This study is based on a validated programme that has been conducted in other countries showing good preventive effect. Based on previous studies, we expect preventive effects of the intervention in the participating adolescents. To ensure that the risks are minimal throughout the programme

For peer review only

implementation and follow-up, we will provide all participants from the pre-intervention phase through post-intervention follow-up, in both control and intervention arms, with axcess to professional help in the form of psychologists, doctors, and nurses if needed. The research group has solid experience implementing the treatment programme in other settings; their experience indicates that the intervention is perceived as positive by students and that participation in the group work has not been stigmatising. All measures and assessments used in the study are validated and tested in many other studies. All researchers and professionals in this study have good pediatric psychiatric competence. They are accustomed to talking with adolescents and will carefully and sensitively handle unpredictable situations. All personnel involved in the study receive training in the intervention. The research team is trained in good clinical practice GCP (Scientific quality, and ethical standards for planning, conducting, recording and reporting trials, involving human participation). The study subjects will never be alone during the intervention, because the intervention is carried out in groups and by trained staff. We will invoke the privacy of group members, and all relevant legislation will be followed regarding use of research data. All members of the research group have undergone training in the intervention by the founders of the programme. The research group consists of General practitioners', psychologists, specialist nurses, and social workers. The trial manager is a specialist psychologist and a PhD, and the Implementation leader is specialist nurse and PhD. The founders of the programme as well as educators are both professors of psychology, one in United states of America and the other one in Iceland.

Results from the study will be presented so that no individuals, including students, parents, and the employer representatives, will be identifiable. All results will be presented on a group level. All participation is voluntary, and the student can withdraw from the study at any time.

The trial will be conducted in compliance with this study protocol, the Declaration of Helsinki and its modifications, and Good Clinical Practice (GCP).

We plan to disseminate the knowledge gained from this study by publishing our results in peer-reviewed scientific journals, giving presentations at national and international conferences, and spreading information through local and national media channels. We will approach local and national politicians to ensure prolongment of the project.

Discussion

The study protocol of this research project has shown how the planning and organisation of the research make it feasible to answer the four research questions.

Question (1). Is the "Thoughts and Health" programme feasible in the context of Swedish schools, from the perspective of the students participating in the programme? The answer will be based on the qualitative interviews and focus group discussions conducted with adolescents participating in the intervention group. As well as analysis of the course surveys gathered after each session and analysis of possible dropouts.

Question (2). Is the "Thoughts and Health" programme feasible in the context of Swedish schools, from the perspective of the primary care professionals leading and supporting the programme implementation? The answer will be based on the analysis of interviews with the professionals who have taken part in focus group discussions or individual interviews after the completion of the intervention course. As well as analysis of the course surveys gathered after each session.

Question (3). Does participation in the "Thoughts and Health" programme have affect on development of depressive symptoms? The answer will be based on collected data and statistical analyses. Outcomes collected before, at and after baseline, with adolescents and adult reports.

For peer review only

Comparisons of effect sizes will be made between the arms of the study (control schools and intervention schools). Measures are described in protocol and table 2.

Question (4). Will the participation in the course “Thoughts and Health” increase the participants' chances of completing middle school with high school eligibility? The answer will be based on follow-up measurements of completion and final grades at graduation from middle school. Comparisons of effect sizes will be made between the arms of the study (control schools and intervention schools).

Current trial status

Recruitment of participants started in September 2019, and the last participant is expected to reach the primary endpoint (18-month follow-up) in April 2021. Primary data analysis will begin in May 2021. The naturalistic follow-up phase of the trial will continue until October 2022.

Patient and public involvement

We will receive input from participants and professionals from focus group discussion and interviews, which will guide the continuation of the intervention study. In the current trial, no participants were involved in the design of the study or in the decision of outcome measures.

Members from the public or students were not involved in designing this study.

We will assess the trial interventions on the participants by collecting information through e-mailed self-rating questionnaire at the end of programme.

For peer review only

Table 1. *Inclusion and exclusion criteria.* *assessed and determined by a psychological assessment, containing the clinical-rated instruments CDI, MINI-KID, RCADS and the children's current status

Inclusion criteria	Exclusion criteria
Eighth grader	Ongoing or past depression
Guardian of eighth grader	Addiction
Understand Swedish language skills, written and oral	Past or current suicidal attempt or ideation
Be able to fill out questionnaires independently	Neuropsychiatric diagnosis that precludes group activities
Completed CDI and be above 75e percentile	Not high enough level of function on the CGAS (cut-off ≥ 61)
Completed psychological assessment	Lacking sufficient Swedish language skills, written and oral
Written and oral consent	Serious mental illness*,
	Ongoing other psychotherapeutic treatment

Table 2. *Instruments and follow-up assessments. Parents o, adolescents x.*

	Screening	Pre Intervention	During treatment	Post-treatment	6-month follow-up	12-month follow-up	18-month follow-up
**Clinician-rated instruments**			**6 weeks**	**3 months**			
MINI-KID		x		x	x	x	x
CGAS		x		x	x	x	x
CGI				x	x	x	x
**Self-rated instruments**							
CDI	x	x	x	x	x	x	x
RCADS		x, o	x	x	x	x	x
EWSAS		x	x	x	x	x	x
MHC-SF		x	x	x	x	x	x
Baseline Questionnaire		x, o		x, o	x, o	x, o	x, o
**Course surveys**			x	x	x	x	x
**Qualitative method**				**3 months**			
Focus group discussions				x			
Individual interviews				x			

Acknowledgement

We would like to thank participating schools. We would like to acknowledge the people who are participating in making this project real. Thank you School Nurses Helena Nordevik Pedersen, Gunilla Börjesson, and Inger Höber, Psychologists Camilla Johansson, Magnus Hjorth, and Henrik Tomasic, Head of Primary care centre in Tjörn Ann-Sofie Lekander, GP Torbjörn Erneholm, and Head of School Health in Tjörn Anna Orvefors. Thanks to Maria EH Larsson.Thanks to Elizabeth Cantor-Graae for language editing. Thanks to Jenna Anderson from Grants Office (Gothia Forum) for help in funding process.

Funding

Social investment grants from Region Västra Götaland, Sweden. Granted: RUN 2018-00614.

Declaration of interest

The author(s) of this paper declare(s) that there is no conflict of interest.

For peer review only

Authors' contributions

All the authors have contributed, are contributing and will contribute to this study, its conductance and publication of study results as follows: Substantial contributions to the conception or design of the work: CW, JL.L, GS, WEC, IM and EÖA. Planning, conduct and reporting of the work: CW, J.L.L, PA, and G.S, Drafting the manuscript: CW and JL.L. Critical revision of the article: all declared authors. Final approval of the version published: all declared authors. Agreement to be accountable for all aspects of the work are resolved: all declared author

References

1. Hankin BL, Abramson LY, Moffitt TE, *et al.* Development of depression from preadolescence to young adulthood: emerging gender differences in a 10-year longitudinal study. *J Abnorm Psychol* 1998;107:128–40. doi:10.1037//0021-843x.107.1.128

2. Lewinsohn PM, Rohde P, Klein DN, *et al.* Natural course of adolescent major depressive disorder: I. Continuity into young adulthood. *J Am Acad Child Adolesc Psychiatry* 1999;38:56–63. doi:10.1097/00004583-199901000-00020

3. Arnarson EO, Craighead WE. Prevention of depression among Icelandic adolescents. *Behav Res Ther* 2009;47:577–85. doi:10.1016/j.brat.2009.03.011

4. Arnarson EO, Craighead WE. Prevention of depression among Icelandic adolescents: a 12-month follow-up. *Behav Res Ther* 2011;49:170–4. doi:10.1016/j.brat.2010.12.008

5. Services S beredning för medicinsk och social utvärdering (SBU); SA for HTA and A of S. Program för att förebygga psykisk ohälsa hos barn. SBU 2010. https://www.sbu.se/sv/publikationer/SBU-utvarderar/program-for-att-forebygga-psykisk-ohalsa-hos-barn/ (accessed 3 Feb 2020).

6. Garber J, Clarke GN, Weersing VR, *et al.* Prevention of depression in at-risk adolescents: a randomized controlled trial. *JAMA* 2009;301:2215–24. doi:10.1001/jama.2009.788

7. Stice E, Rohde P, Seeley JR, *et al.* Brief Cognitive-Behavioral Depression Prevention Program for High-Risk Adolescents Outperforms Two Alternative Interventions: A Randomized Efficacy Trial. *J Consult Clin Psychol* 2008;76:595. doi:10.1037/a0012645

8. Clarke GN, Hawkins W, Murphy M, *et al.* Targeted prevention of unipolar depressive disorder in an at-risk sample of high school adolescents: a randomized trial of a group cognitive intervention. *J Am Acad Child Adolesc Psychiatry* 1995;34:312–21. doi:10.1097/00004583-199503000-00016

9. Erik H. Erikson. Identity, youth and crisis. New York: WW Norton Company, 1968

10. Gillham JE, Hamilton J, Freres DR, et al. Preventing depression among early adolescents in the primary care setting: a randomized controlled study of the Penn Resiliency Program. *J Abnorm Child Psychol* 2006;34:203–19. doi:10.1007/s10802-005-9014-7

11. Seligman MEP, Schulman P, Tryon AM. Group prevention of depression and anxiety symptoms. *Behav Res Ther* 2007;45:1111–26. doi:10.1016/j.brat.2006.09.010

12. Stice E, Burton E, Bearman SK, et al. Randomized trial of a brief depression prevention program: an elusive search for a psychosocial placebo control condition. *Behav Res Ther* 2007;45:863–76. doi:10.1016/j.brat.2006.08.008

For peer review only

13. Horowitz JL, Garber J. The prevention of depressive symptoms in children and adolescents: A meta-analytic review. *J Consult Clin Psychol* 2006;74:401–15. doi:10.1037/0022-006X.74.3.401

14. Merry S, McDowell H, Hetrick S, et al. Psychological and/or educational interventions for the prevention of depression in children and adolescents. *Cochrane Database Syst Rev* 2004;:CD003380. doi:10.1002/14651858.CD003380.pub2

15. Roberts CM, Kane R, Bishop B, et al. The prevention of anxiety and depression in children from disadvantaged schools. *Behav Res Ther* 2010;48:68–73. doi:10.1016/j.brat.2009.09.002

16. Sawyer MG, Pfeiffer S, Spence SH, et al. School-based prevention of depression: a randomised controlled study of the beyondblue schools research initiative. *J Child Psychol Psychiatry* 2010;51:199–209. doi:10.1111/j.1469-7610.2009.02136.x

17. Klein DN, Glenn CR, Kosty DB, *et al.* Predictors of first lifetime onset of major depressive disorder in young adulthood. *J Abnorm Psychol* 2013;122:1–6. doi:10.1037/a0029567

18. Chan A-W, Tetzlaff JM, Altman DG, Laupacis A, Gøtzsche PC, Krleža-Jerić K, Hróbjartsson A, Mann H, Dickersin K, Berlin J, Doré C, Parulekar W, Summerskill W, Groves T, Schulz K, Sox H, Rockhold FW, Rennie D, Moher D. SPIRIT 2013 Statement: Defining standard protocol items for clinical trials. Ann Intern Med. 2013;158(3):200-207

19. Ivarsson T, Svalander P, Litlere O. The Children's Depression Inventory (CDI) as measure of depression in Swedish adolescents. A normative study. *Nordic Journal of Psychiatry*, 60 (3) (2006), pp. 220-226

20. Sheehan, D.V.; Sheehan, K.H.; Shyte, R.D.; Janavs, J.; Bannon, Y.; Rogers, J.E.; Milo, K.M.; Stock, S.L.; Wilkinson, B. Reliability and validity of the Mini International Neurpsychiatric Interview for Children and Adolescents (MINI-KID). *J. Clin. Psychiatry* 2010, 71, 313–326.

21. Chorpita, B. F., Moffitt, C., & Gray, J. (2005). Psychometric properties of the Revised Child Anxiety and Depression Scale in a clinical sample. *Behaviour Research and Therapy*, 43, 309-322.

22. American Psychiatric Association. (2013). Diagnostic and statistical manual of mental disorders (5th ed.). Washington, DC: Author.

23. Shaffer, D., Gould, M.S., Brasic, J., Ambrosini, P., Fisher, P., Bird, H., & Aluwahlia, S. (1983). A Children's Global Assessment Scale (CGAS). *Archives of General Psychiatry*, 40, 1228-1231.

24. Mundt, J.C., Marks, I.M., Shear K.M., Greist J., (2002). Impairment in functioning. The work and social adjustment scale: a simple measure of impairment of functioning. *The British Journal of Psychiatry*, 180, 461-464.

25. Keyes, C. L. M. (2009). Atlanta: Brief description of the mental health continuum short form (MHC-SF). Available: https://www.aacu.org/sites/default/files/MHC-SFEnglish.pdf. [On–line, retrieved 2020-03-30]

26. Keyes CLM, Wissing M, Potgieter JP, *et al.* Evaluation of the mental health continuum-short form (MHC-SF) in setswana-speaking South Africans. *Clin Psychol Psychother* 2008;15:181–92. doi:10.1002/cpp.572

27. Keyes CLM. The Subjective Well-Being of America's Youth: Toward a Comprehensive Assessment. *Adolescent & Family Health* 2006;4:3–11.

For peer review only

28. Lamers SMA, Glas CAW, Westerhof GJ, *et al.* Longitudinal evaluation of the mental health continuum-short form (MHC-SF): Measurement invariance across demographics, physical illness, and mental illness. *Eur J Psychol Assess* 2012;28:290–6. doi:10.1027/1015-5759/a000109

For peer review only

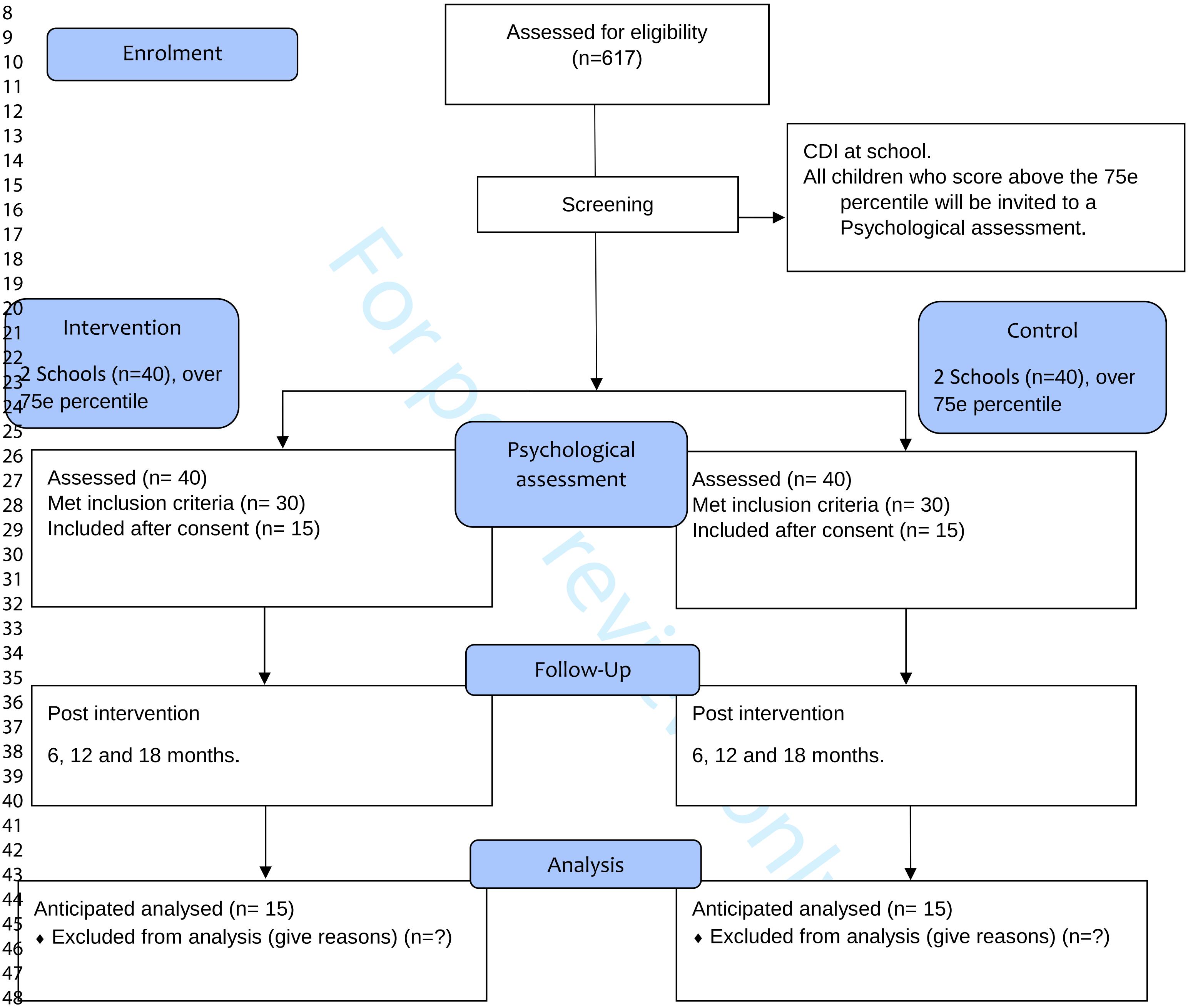
Enrolment
Assessed for eligibility (n=617)
Screening
CDI at school.
All children who score above the 75e percentile will be invited to a Psychological assessment.
Intervention
2 Schools (n=40), over 75e percentile
Control
2 Schools (n=40), over 75e percentile
Psychological assessment
Assessed (n= 40)
Met inclusion criteria (n= 30)
Included after consent (n= 15)
Assessed (n= 40)
Met inclusion criteria (n= 30)
Included after consent (n= 15)
Follow-Up
Post intervention
6, 12 and 18 months.
Post intervention
6, 12 and 18 months.
Analysis
Anticipated analysed (n= 15)
♦ Excluded from analysis (give reasons) (n=?)
Anticipated analysed (n= 15)
♦ Excluded from analysis (give reasons) (n=?)

Reporting checklist for protocol of a clinical trial.

Based on the SPIRIT guidelines.

Instructions to authors

Complete this checklist by entering the page numbers from your manuscript where readers will find each of the items listed below.

Your article may not currently address all the items on the checklist. Please modify your text to include the missing information. If you are certain that an item does not apply, please write "n/a" and provide a short explanation.

Upload your completed checklist as an extra file when you submit to a journal.

In your methods section, say that you used the SPIRITreporting guidelines, and cite them as:

Chan A-W, Tetzlaff JM, Altman DG, Laupacis A, Gøtzsche PC, Krleža-Jerić K, Hróbjartsson A, Mann H, Dickersin K, Berlin J, Doré C, Parulekar W, Summerskill W, Groves T, Schulz K, Sox H, Rockhold FW, Rennie D, Moher D. SPIRIT 2013 Statement: Defining standard protocol items for clinical trials. Ann Intern Med. 2013;158(3):200-207

For peer review only

		Reporting Item	Page Number
**Administrative information**			
Title	#1	Descriptive title identifying the study design, population, interventions, and, if applicable, trial acronym	1

Trial registration	#2a	Trial identifier and registry name. If not yet registered, name of intended registry	1
Trial registration: data set	#2b	All items from the World Health Organization Trial Registration Data Set	n/a
Protocol version	#3	Date and version identifier	n/a
Funding	#4	Sources and types of financial, material, and other support	11
Roles and responsibilities: contributorship	#5a	Names, affiliations, and roles of protocol contributors	1
Roles and responsibilities: sponsor contact information	#5b	Name and contact information for the trial sponsor	n/a
Roles and responsibilities: sponsor and funder	#5c	Role of study sponsor and funders, if any, in study design; collection, management, analysis, and interpretation of data; writing of the report; and the decision to submit the report for publication, including whether they will have ultimate authority over any of these activities	n/a
Roles and responsibilities: committees	#5d	Composition, roles, and responsibilities of the coordinating centre, steering committee, endpoint adjudication committee, data management team, and	n/a

For peer review only

		other individuals or groups overseeing the trial, if applicable (see Item 21a for data monitoring committee)	
**Introduction**			
Background and rationale	#6a	Description of research question and justification for undertaking the trial, including summary of relevant studies (published and unpublished) examining benefits and harms for each intervention	2
2Background and rationale: choice of comparators	#6b	Explanation for choice of comparators	2
Objectives	#7	Specific objectives or hypotheses	2
Trial design	#8	Description of trial design including type of trial (eg, parallel group, crossover, factorial, single group), allocation ratio, and framework (eg, superiority, equivalence, non-inferiority, exploratory)	3
**Methods: Participants, interventions, and outcomes**			
Study setting	#9	Description of study settings (eg, community clinic, academic hospital) and list of countries where data will be collected. Reference to where list of study sites can be obtained	3

For peer review only

Eligibility criteria	#10	Inclusion and exclusion criteria for participants. If applicable, eligibility criteria for study centres and individuals who will perform the interventions (eg, surgeons, psychotherapists)	3
Interventions: description	#11a	Interventions for each group with sufficient detail to allow replication, including how and when they will be administered	7
Interventions: modifications	#11b	Criteria for discontinuing or modifying allocated interventions for a given trial participant (eg, drug dose change in response to harms, participant request, or improving / worsening disease)	n/a
Interventions: adherance	#11c	Strategies to improve adherence to intervention protocols, and any procedures for monitoring adherence (eg, drug tablet return; laboratory tests)	n/a
Interventions: concomitant care	#11d	Relevant concomitant care and interventions that are permitted or prohibited during the trial	n/a
Outcomes	#12	Primary, secondary, and other outcomes, including the specific measurement variable (eg, systolic blood pressure), analysis metric (eg, change from baseline, final value, time to event), method of aggregation (eg, median, proportion), and time point for each outcome. Explanation of the clinical relevance of chosen efficacy and harm outcomes is strongly recommended	5

For peer review only

Participant timeline	#13	Time schedule of enrolment, interventions (including any run-ins and washouts), assessments, and visits for participants. A schematic diagram is highly recommended (see Figure)	10
Sample size	#14	Estimated number of participants needed to achieve study objectives and how it was determined, including clinical and statistical assumptions supporting any sample size calculations	7
Recruitment	#15	Strategies for achieving adequate participant enrolment to reach target sample size	4
**Methods: Assignment of interventions (for controlled trials)**			
Allocation: sequence generation	#16a	Method of generating the allocation sequence (eg, computer-generated random numbers), and list of any factors for stratification. To reduce predictability of a random sequence, details of any planned restriction (eg, blocking) should be provided in a separate document that is unavailable to those who enrol participants or assign interventions	n/a
Allocation concealment mechanism	#16b	Mechanism of implementing the allocation sequence (eg, central telephone; sequentially numbered, opaque,	n/a

For peer review only

		sealed envelopes), describing any steps to conceal the sequence until interventions are assigned	
Allocation: implementation	#16c	Who will generate the allocation sequence, who will enrol participants, and who will assign participants to interventions	n/a
Blinding (masking)	#17a	Who will be blinded after assignment to interventions (eg, trial participants, care providers, outcome assessors, data analysts), and how	n/a
Blinding (masking): emergency unblinding	#17b	If blinded, circumstances under which unblinding is permissible, and procedure for revealing a participant's allocated intervention during the trial	n/a
**Methods: Data collection, management, and analysis**			
Data collection plan	#18a	Plans for assessment and collection of outcome, baseline, and other trial data, including any related processes to promote data quality (eg, duplicate measurements, training of assessors) and a description of study instruments (eg, questionnaires, laboratory tests) along with their reliability and validity, if known. Reference to where data collection forms can be found, if not in the protocol	10

For peer review only

Data collection plan: retention	#18b	Plans to promote participant retention and complete follow-up, including list of any outcome data to be collected for participants who discontinue or deviate from intervention protocols	n/a
Data management	#19	Plans for data entry, coding, security, and storage, including any related processes to promote data quality (eg, double data entry; range checks for data values). Reference to where details of data management procedures can be found, if not in the protocol	7
Statistics: outcomes	#20a	Statistical methods for analysing primary and secondary outcomes. Reference to where other details of the statistical analysis plan can be found, if not in the protocol	7
Statistics: additional analyses	#20b	Methods for any additional analyses (eg, subgroup and adjusted analyses)	n/a
Statistics: analysis population and missing data	#20c	Definition of analysis population relating to protocol non-adherence (eg, as randomised analysis), and any statistical methods to handle missing data (eg, multiple imputation)	n/a

Methods: Monitoring

Data monitoring: formal committee	#21a	Composition of data monitoring committee (DMC); summary of its role and reporting structure; statement of whether it is independent from the sponsor and	n/a

For peer review only

		competing interests; and reference to where further details about its charter can be found, if not in the protocol. Alternatively, an explanation of why a DMC is not needed	
Data monitoring: interim analysis	#21b	Description of any interim analyses and stopping guidelines, including who will have access to these interim results and make the final decision to terminate the trial	n/a
Harms	#22	Plans for collecting, assessing, reporting, and managing solicited and spontaneously reported adverse events and other unintended effects of trial interventions or trial conduct	8
Auditing	#23	Frequency and procedures for auditing trial conduct, if any, and whether the process will be independent from investigators and the sponsor	n/a
**Ethics and dissemination**			
Research ethics approval	#24	Plans for seeking research ethics committee / institutional review board (REC / IRB) approval	8
Protocol amendments	#25	Plans for communicating important protocol modifications (eg, changes to eligibility criteria, outcomes, analyses) to relevant parties (eg, investigators, REC / IRBs, trial participants, trial registries, journals, regulators)	n/a

For peer review only

Consent or assent	#26a	Who will obtain informed consent or assent from potential trial participants or authorised surrogates, and how (see Item 32)	8
Consent or assent: ancillary studies	#26b	Additional consent provisions for collection and use of participant data and biological specimens in ancillary studies, if applicable	n/a
Confidentiality	#27	How personal information about potential and enrolled participants will be collected, shared, and maintained in order to protect confidentiality before, during, and after the trial	n/a
Declaration of interests	#28	Financial and other competing interests for principal investigators for the overall trial and each study site	11
Data access	#29	Statement of who will have access to the final trial dataset, and disclosure of contractual agreements that limit such access for investigators	7
Ancillary and post trial care	#30	Provisions, if any, for ancillary and post-trial care, and for compensation to those who suffer harm from trial participation	
Dissemination policy: trial results	#31a	Plans for investigators and sponsor to communicate trial results to participants, healthcare professionals, the public, and other relevant groups (eg, via publication, reporting in results databases, or other data sharing arrangements), including any publication restrictions	n/a

For peer review only

Dissemination policy: authorship	#31b	Authorship eligibility guidelines and any intended use of professional writers	n/a
Dissemination policy: reproducible research	#31c	Plans, if any, for granting public access to the full protocol, participant-level dataset, and statistical code	n/a

Appendices

Informed consent materials	#32	Model consent form and other related documentation given to participants and authorised surrogates	attached
Biological specimens	#33	Plans for collection, laboratory evaluation, and storage of biological specimens for genetic or molecular analysis in the current trial and for future use in ancillary studies, if applicable	n/a

None The SPIRIT checklist is distributed under the terms of the Creative Commons Attribution License CC-BY-ND 3.0. This checklist can be completed online using https://www.goodreports.org/, a tool made by the EQUATOR Network in collaboration with Penelope.ai

For peer review only

BMJ Open

Is the "Thoughts and Health" programme feasible in the context of Swedish schools, a quasi-experimental controlled trial - Study Protocol

Journal:	*BMJ Open*
Manuscript ID	bmjopen-2020-040374.R2
Article Type:	Protocol
Date Submitted by the Author:	07-Dec-2020
Complete List of Authors:	Wikberg, Carl; University of Gothenburg Institute of Medicine, School of Public Health and Community Medicine<br>Augustsson, Pia; University of Gothenburg Department of Public Health and Community Medicine; Vastra Gotaland Primary Health Care, Närhälsan Research and Development Primary Health Care, Region Västra Götaland, Gothenburg, Sweden<br>Sveinsdottir, Gudny; Vastra Gotaland Primary Health Care, Närhälsan Research and Development Primary Health Care, Region Västra Götaland, Gothenburg, Sweden<br>Craighead, Edward; Emory University, Department of Psychology, Psychiatry & Behavioral Sciences<br>Arnarson, Erikur<br>Marteinsdottir, Ina; University of Kalmar, Department of Medicine and Optometry<br>Lilja, Josefine L.; University of Gothenburg Department of Psychology, Department of Psychology, University of Gothenburg, Gothenburg, Sweden
**Primary Subject Heading**:	Mental health
Secondary Subject Heading:	General practice / Family practice, Public health, Mental health
Keywords:	Child & adolescent psychiatry < PSYCHIATRY, PUBLIC HEALTH, Depression & mood disorders < PSYCHIATRY

SCHOLARONE™
Manuscripts

I, the Submitting Author has the right to grant and does grant on behalf of all authors of the Work (as defined in the below author licence), an exclusive licence and/or a non-exclusive licence for contributions from authors who are: i) UK Crown employees; ii) where BMJ has agreed a CC-BY licence shall apply, and/or iii) in accordance with the terms applicable for US Federal Government officers or employees acting as part of their official duties; on a worldwide, perpetual, irrevocable, royalty-free basis to BMJ Publishing Group Ltd ("BMJ") its licensees and where the relevant Journal is co-owned by BMJ to the co-owners of the Journal, to publish the Work in this journal and any other BMJ products and to exploit all rights, as set out in our licence.

The Submitting Author accepts and understands that any supply made under these terms is made by BMJ to the Submitting Author unless you are acting as an employee on behalf of your employer or a postgraduate student of an affiliated institution which is paying any applicable article publishing charge ("APC") for Open Access articles. Where the Submitting Author wishes to make the Work available on an Open Access basis (and intends to pay the relevant APC), the terms of reuse of such Open Access shall be governed by a Creative Commons licence – details of these licences and which Creative Commons licence will apply to this Work are set out in our licence referred to above.

Other than as permitted in any relevant BMJ Author's Self Archiving Policies, I confirm this Work has not been accepted for publication elsewhere, is not being considered for publication elsewhere and does not duplicate material already published. I confirm all authors consent to publication of this Work and authorise the granting of this licence.

For peer review only

Is the "Thoughts and Health" programme feasible in the context of Swedish schools, a quasi-experimental controlled trial - Study Protocol

Carl Wikberg[1,2], Pia Augustsson[1,2], Gudny Sveinsdottir[2] , W Edward Craighead[3], Eirikur Örn Arnarson[4,5], Ina Marteinsdottir[6], Josefine L Lilja[2,7].

Affiliation:

1 School of Public Health and Community Medicine, Institute of Medicine, Sahlgrenska Academy, University of Gothenburg, Gothenburg, Sweden.

2 Närhälsan Research and Development Primary Health Care, Region Västra Götaland, Gothenburg, Sweden.

3 Department of Psychiatry and Behavioral Sciences and the Department of Psychology, Emory University, 12 Executive Park Drive NE, Suite 200, Atlanta, GA 30329, USA.

4 Department of Psychiatry, Landspítali-University Hospital, Reykjavík, Iceland.

5 Faculty of Medicine, School of Health Sciences, Reykjavík, Iceland

6 Department of Clinical and Experimental Medicine, Center for Social and Affective Neuroscience, Linköping University, 581 85, Linköping, Sweden.

7 Department of Psychology, University of Gothenburg, Gothenburg, Sweden.

Corresponding author

Carl Wikberg – carl.wikberg@allmed.gu.se

Box 428, 405 30 Gothenburg

+46703821660

Keywords: Depression, Adolescence, Prevention, Feasibility

Word count: 4876

For peer review only

Abstract

Introduction

Clinical depression is a substantial problem among adolescents, increasing significantly at about age 15. It causes impairment in social, academic, and familial relationships as well as ongoing cognitive and emotional difficulties for the individual. A study in Iceland demonstrated that a cognitive-behavioural, developmentally based intervention programme “Thoughts and Health” prevented initial episodes of depression and/or dysthymia (MDD/DYS) in adolescents for up to 12 months following completion of the programme. We would like to test the feasibility of implementing the Icelandic method in a Swedish context and to evaluate the long-term effects of such a programme.

Methods and analysis

A quasi-experimental controlled design combined with qualitative and quantitative methods will be used to address the research questions.

In this study 617 children aged ~14 will be screened for depression, and those “at risk” for development of clinical depression will be offered a 12 week course “Thoughts and Health”. This course aims to prevent first depression in adolescents. A comparable group of children will function as controls.

Depending on type of variable, baseline comparisons between the two groups of relevant initial measures will be evaluated with t-tests or chi-square analyses. The effects of the programme on the development of clinical levels of depression will be evaluated using the 6-, 12-, and 18-month follow-up data. Index parental depression at baseline will be tested as a moderator in the evaluation of the prevention programme effects.

Ethics and dissemination.

This study is approved by the Swedish Ethical Review Board (reference number 2019-03347) in Gothenburg.

We plan to disseminate the knowledge gained from this study by publishing our results in peer-reviewed scientific journals and other scholarly outlets.

Registration details: NCT04128644 ClinicalTrials.gov

For peer review only

Strengths and limitations of this study

- Quasi-experimental controlled design will provide information about whether the intervention prevents depressive episodes in adolescence in a Swedish sample.
- Qualitative data will shed light on how the intervention was perceived by the adolescents and how the personnel experienced the implementation process.
- Quantitative data will provide information regarding adolescents' mental health and social functioning.
- Follow-up assessments will provide information regarding the intervention's long-term effects on school performance and mental health.
- The study's lack of a randomised controlled design and the few participants limit its generalisability.

For peer review only

Introduction

Clinical depression is a substantial problem among adolescents, increasing significantly at about age 15. It causes impairment in social, academic, and familial relationships as well as ongoing cognitive and emotional difficulties for the individual [1, 2].

A study in Iceland demonstrated that a cognitive-behavioural, developmentally based intervention programme called “Thoughts and Health” prevented initial episodes of depression and/or dysthymia (MDD/DYS) among Icelandic adolescents for up to 12 months following completion of the programme [3,4]. Other studies have demonstrated that similar programmes can be employed to prevent depression among teenagers who have never been depressed and/or who previously have been depressed [5–8]. The “Thoughts and Health” programme utilises principles of both behavioural (e.g., relaxation, behavioural activation (assertion and social approach)) and cognitive (e.g., identification and modification of irrational thoughts, thinking errors, and self-beliefs) interventions. The programme procedures were specifically adapted to the appropriate developmental level of the youth participants, with developmental level defined as Stage 5 (Identity vs Confusion) of Erikson’s conceptual model of human psychosocial development [9]. Specifically, Stage 5 encompasses adolescence, during which individuals *develop a sense of self* within the context of emerging independence from the family and expansion of interpersonal/social relationships.

However, despite the positive impact of such programmes, preventive effects have not been consistently obtained [10–12]. Reviews have concluded that universal prevention programmes (programmes applied to the general population) have not been effective in preventing depression (see [13, 14]), and excellently conducted studies in Australia have provided data consistent with that conclusion [15, 16]. In general, only targeted programmes (*selective* programmes – for populations exposed to risk factors or *indicated* programmes – for populations who are experiencing some symptoms) have shown significant short-term effects in preventing depression, e.g., [1, 5–7].

Most programmes aimed at preventing depression have included both youth who have suffered a prior depression and those who have never been depressed. The study of prevention of relapse or recurrence in prevention programmes for those who have already suffered a major depressive disorder (MDD) is clearly useful, but earlier studies have not distinguished between prevention of the first episode of MDD and the prevention of relapse/recurrence of MDD. However, because of the recurrent nature of the disorder and the differing causal factors and probability patterns of first versus subsequent episodes [17] and in order to prevent the deleterious effects of an episode of MDD in youth, it is especially important to study the prevention of the first or initial episode among never previously depressed subjects.

Since clinical depression among adolescents increases significantly at about age 15, and Swedish children finish their compulsory school (junior high school) at that age, being at risk for depression can be a reason for failing their final examinations, thereby limiting their chances for further academic studies.

We would like to test the feasibility of implementing the Icelandic method in a Swedish context and to evaluate the long-term effects of such a programme. We would like to test the method with a quasi-experimental controlled trial and to evaluate the outcomes after 6, 12 and 18 months. The control group will be assessment-only adolescents; they receive ordinary “Student Health”, they participate in all study assessments, but they are not offered the intervention, “Thoughts and Health”.

This is, to our knowledge, the first study in Sweden that employs a longitudinal design to evaluate the effects of a school-based programme aimed at preventing depression in adolescents.

For peer review only

This study will be implemented together and within the municipalities of Tjörn and Orust, two islands off the west coast of Sweden, with each island only having two middle schools. Thus, this study is built on a close collaboration between academia, primary care, and the community.

We are hopeful that this programme will be transferrable to a Swedish context and that it will be possible to further develop and enhance the method. Although Iceland and Sweden are similar in many ways, the school system and cultural context differ to some extent.

We hypothesise that programme implementation will reduce the risk of developing depressive symptoms among participating students compared to their control counterparts, and we will examine this in participating adolescents up to 18 months after programme completion. We also hypothesise that adolescents in the intervention group who participate in the programme will finish school with complete grades to a greater extent (more students complete their schooling) than the control group.

Aim

This study aims to test the feasibility of implementing an Icelandic cognitive-behavioural programme designed to prevent depression, “Thoughts and Health” [3,4], in a Swedish school setting.

The perspectives of both the participating adolescents and the primary care professionals who lead and support the programme in the schools will be taken into account when evaluating the programme’s feasibility.

The study also aims to evaluate the number of students who finish junior high school (ninth grade, ~15 years old) with complete grades. In this study, the outcomes concerning complete grades of the students participating in the intervention programme will be compared to the complete grades of those in the control group.

Our primary research questions are:

Is the “Thoughts and Health” programme feasible in the context of Swedish schools, from the perspective of the students participating in the programme?

Is the “Thoughts and Health” programme feasible in the context of Swedish schools, from the perspective of the primary care professionals leading and supporting the programme implementation?

Our secondary research questions are:

Does participation in the “Thoughts and Health” programme affect the number of students developing depressive symptoms?

Will participation in the course “Thoughts and Health” increase the participants' chances of completing compulsory school with high school eligibility?

Method

Study design

A quasi-experimental controlled design combined with qualitative and quantitative methods will be used to address the research questions. NCT04128644, Clinicaltrials.com

We have used the SPIRIT reporting guidelines [18]

Participants

An invitation regarding participation in the study to high schools in the area resulted in four junior high schools from the municipalities of Tjörn (Häggvall and Bleket, intervention arm) and Orust (Henån och Ängås, control arm) that will participate in the study. The allocation of the schools was

For peer review only

chosen on the basis of their rural location and socioeconomic similarity, willingness to participate, and available staff. Since all schools had shown interest in participating in this study, the assignment of intervention and control arms was determined on the basis of available resources; therefore, Tjörn was chosen for the intervention programme at this stage. Two intervention courses are planned (I and II), with course I beginning at the start of the fall semester (~1st September) 2019 and course II beginning at the start of the fall semester (~1st September) 2020.

In total, approximately 320 eighth grade Swedish adolescents aged 14 years will participate in a pre-intervention screening process per course. In the pre-intervention screening process, all students will complete the Children's Depression Inventory (CDI, described below) [19] assessment instrument via an online questionnaire tool. This will be part of the existing "Student Health" ordinary assignments that include health and mental health status checkups as per the Swedish School Law (Skollagen 2010:800).

We are aware of the small number of students that will be part of the collected data at this early stage, and we are planning a full scale randomised trial once we have completed this first study; hence, there were no power calculations of sample size at this stage.

All students who score at or above the 75th percentile on the CDI screening will be qualified initially for possible inclusion in the study (expected n=80 total per course). They will receive an invitation together with their parents to attend a psychological assessment interview (the Mini International Neuropsychiatric Interview for Children and Adolescents ((MINI-KID), described below) [20]. At the interview, they will be given the opportunity to discuss their CDI result and if needed, to be channelled to the right level of care. (Example: If a student scored very high on CDI, the psychologist will need to decide whether there is any underlying diagnosis that requires treatment or if the student has any other condition that also requires immediate attention and treatment.) If a student is diagnosed with depression at this phase, he or she will no longer be eligible to participate in the study and will be directed to appropriate mental health care resources in the community. Prior to attending this psychological assessment, students and their parents will separately fill out the Revised Children's Anxiety and Depression Scale (RCADS) [21] assessment instrument.

Based on the MINI-KID results, students who meet criteria for having any current or past major depression, dysthymia, bipolar disorder I or II, cyclothymia, anorexia, bulimia, psychotic disorder, alcohol or substance dependence, attention deficit hyperactivity disorder (ADHD), oppositional defiant disorder (ODD), or conduct disorder will be excluded. All other students thus fulfill inclusion criteria. All students, both in the intervention and control arms, will be invited to participate in the study via an appropriately worded letter (expected n=40 total per study arm). See Table 1 for inclusion and exclusion criteria.

Thus, about 20 students per course will be offered to participate in the intervention programme. These students will be approached by the programme staff and offered the opportunity to participate in the baseline clinical interview and assessment, the intervention programme, and post-intervention follow-up assessments at 6, 12, and 18 months after programme completion (Figure 1).

Participants in the intervention and control arm will be offered tickets to the cinema as a gesture of gratitude for their participation in each of the follow-up assessments.

Measures and assessments

Measures and assessments are described below. The instruments, persons involved and the time point for each measure or assessment are shown in Table 2. All psychometric measures are administered by esMaker (htpps://entergate.se/products/esmaker/). Course surveys are distributed in the form of hard copies. If participant frequency is shown to be low, those who have not completed the measures will receive a reminder by email.

For peer review only

Outcomes

CDI, ordinal variables (collected during pre-intervention phase, at course start, and at 6-, 12-, and 18-months follow-up). The CDI is a 27-item self-reported, symptom-oriented scale that rates the severity of depression during the past month. It is designed for children/adolescents, ages 7-17 years. Each of the 27 items comprises 3 sentences used by the student to rate the severity of the symptoms. This inventory has previously shown excellent internal consistency (Cronbach alpha coefficients of .83 to .94). The present study utilises the total score of the Swedish version of the CDI [19]. The CDI will be used both in screening and in follow-ups with participating children.

The Revised Children's Anxiety and Depression Scale (RCADS), ordinal variables (collected during pre-intervention phase and at 6-, 12-, and 18-months follow-up). RCADS is a widely used questionnaire designed to assess clinical syndromes of anxiety as well as depression, based on the DSM-IV criteria. The RCADS provides two total scores and has 6 subscales: Separation Anxiety Disorder (SAD), Social Phobia (SoP), Obsessive Compulsive Disorder (OCD), Panic Disorder (PD), Generalised Anxiety Disorder (GAD), and Major Depressive Disorder (MDD). The internal consistency of the RCADS subscales is high, with Cronbach alphas ranging from 0.78 to 0.88. There are two versions of RCADS; one for adolescents (RCADS) and one for parents (RCADS-P).

MINI-KID, categorical variables (collected during pre-intervention phase and at 6-, 12-, and 18-months follow-up). The MINI-KID is a structured interview for children and young people aged 4-17 years old [20]. It is based on diagnoses using the Diagnostic and Statistical Manual of Mental Disorders (DSM-5) [22] and the tenth edition of International Statistical Classification of Diseases and Related Health Problems (ICD-10). Both parents and youths will participate in the interview, but the interview can also be conducted with only young people. The interview aims to ensure diagnosis and inclusion/exclusion criteria.

Demographic information and somatic health, categorical variables (collected at course start and at 6-, 12-, and 18-months follow-up). The baseline questionnaire will include items regarding sociodemographics, physical activity, drug use, and some questions about mobile and internet usage.

Children's Global Assessment Scale (CGAS)[23], ordinal variables (collected during pre-intervention phase). The CGAS will be used to measure global functioning. The assessment will be made by the person who conducts the MINI-KID interview. The CGAS estimate is made on a scale of 1-100, where a higher score indicates a higher functional level. This will be completed for participating children.

Education Work and Social Adjustment Scale (EWSAS) [24], ordinal variables (collected at course start, course middle (6 weeks after course start), and at 6-, 12-, and 18-months follow-up). The EWSAS measures general level of function. It is an adaptation of the Work and Social Adjustment Scale (WSAS) and measures the level of function in five areas: relationships, family, leisure time, social activities, and school and everyday activities. This will be completed by participating children.

Mental Health Continuum – Short Form (MHC – SF), ordinal variables (collected at course start, course middle (6 weeks after course start), and at 6-, 12-, and 18-months follow-up). The MHC-SF is an increasingly used questionnaire that measures three components of well-being: emotional, social, and psychological. It has shown excellent internal consistency (>.80) and discriminant validity in adolescents (ages 12-18 years) and adults in the U.S., in the Netherlands, and in South Africa [25-28]. MHC-SF consists of 14 items asking about the frequency of well-being experiences during the last month. There are three items concerning emotional well-being such as feelings of being happy, interested in life and satisfied, five items concerning social well-being, reference, actualisation, contribution, coherence and integration, and six items concerning psychological well-being capturing purpose in life, environmental mastery, autonomy, personal growth, positive relations and self-acceptance. Answers are given on a 6-point Likert scale from 0 = never, 1 = once or twice, 2 = about

For peer review only

once a week, 3 = about 2 or 3 times a week, 4 = almost every day, 5 = every day. Cronbach's alpha was 0.92 for emotional well-being, 0.88 for social well-being and 0.92 for psychological well-being. Scores on each well-being component are summed and categorised as "flourishing", "moderately healthy" or "languishing" according to official MHC-SF guidelines. The MHC-SF has been useful in improving the scientific understanding of the risk of future mental illness (major depressive episode, GAD, panic attacks, risk of premature mortality, healthcare utilisation, missed days of work, suicidality, and self-reported academic impairment among college students). The MHC-SF will be completed by participating children.

Parental depression, binary variable (collected during pre-intervention phase). At the time of the baseline interview, adolescents will be asked if their parents previously have suffered from or currently suffer from depression. Similarly, parents will be asked if they have previously suffered from or currently suffer from depression. These data are collected because there are known associations between depression in parents and the health of their children (6). We would like to know if any of the parents have had or currently have a depressive episode as that may be related to the depressive outcomes in their children. Both adolescents and parents data will be analysed separately. This will be useful for further analysis regarding long-term follow ups. As an example, we will be able to see whether intervention group and control group parents are similar regarding possible previous or current state of depression.

Course surveys, ordinal variables (collected after each course session). After each course session (total of 12 sessions per course), the students and the primary care professionals leading and supporting the course will directly provide their feedback about the course session by answering a short survey. Different surveys will be provided to students and the professionals, respectively. These surveys will assess feasibility, satisfaction and overall impression of the session.

Course attendance and dropouts, binary variables (collected during each course session). Attendance and dropouts will be recorded at each course meeting, as a measure of the feasibility and acceptance of the programme.

Focus groups and interviews, qualitative analysis (collected within 1 month after course end and at 18-months follow-up). All participants (students and professionals) will be invited to take part in focus group discussions or individual interviews after the completion of the full course. These discussions will be held separately, with students in one or two groups and the professionals in another, or as individual interviews. Focus groups will be held with the students and individual interviews with professionals.

Final junior high school grades, categorical variables (collected at approximately 18 months after course end). Students' final grades in junior high school (that is, in ninth grade, or 18 months after their inclusion in the programmes) will be collected to be able to evaluate the number of students who finish school with complete grades. The information will be provided by the school administration upon consent (supplementary ____________file) from the students.

All psychological assessments will be conducted by a trained psychologist from the Primary Care Centre, and the focus groups and interviews will be conducted by the authors CW (PhD and Specialist nurse) and PA (PhD student).

Data management

A research journal will be used to follow the person during the programme and follow-up period. The research journal will be kept at the primary care R&D unit, Kungsgatan, in Gothenburg, where data from the research journal are entered. Data in the database at the research unit will only be entered with code numbers. Only the research leaders will then have access to the code key, which is stored in in a locked safe at the research unit. The research journals will be kept at the research unit and will be

For peer review only

anonymised during the evaluation phase . The composition of the research group may change during the course of the project, but only authorised persons who are part of the research group will have access to data. The usual privacy is maintained.

Intervention: Prevention Programme

The prevention programme will be based on the Icelandic "Thoughts and Feelings" programme [1] and will include 12 group sessions of 90-minutes duration, delivered twice per week for two weeks and then once a week for eight weeks in the school setting by "Student Health" school psychologists. School nurses will also attend each session as support for the students. The programme uses two intervention manuals: a group leader manual and a student manual. Both of these manuals have been translated first from Icelandic to English, and then from the English version into Swedish, and finally corrected for language in the Swedish version. After translation, the manuals were additionally reviewed by several eighth-grade students for cultural acceptability (e.g., word choice) and adapted as per their feedback. The course material is based on cognitive behaviour theory, and the course will be held as a group activity. The course was originally developed as a group activity, and we will test this method in our study, as part of the evaluation of whether the method is suitable for Swedish adolescents of today. The course contains group sessions focusing on a specific topic, after which they will describe their state of health and feelings in a diary and receive some home assignments to be completed between sessions. All sessions start with a light refreshment and a summary of the previous session. Session #1 is an introduction. Session #2 is about "the spiral", that is, how negative or positive thoughts and actions affect subsequent actions and feelings. Session #3 is about relaxation. Session #4 will discuss the concept of a "baseline". The remaining sessions deal with the following topics: setting targets (#5), feelings (#6), negative thoughts (#7), irrational thoughts (#8), self-perception (#9), communication (#10), stop and reconsideration (#11) and course consummation (#12, final session).

Statistical Analysis

Depending on type of variable, baseline comparisons of the two groups of relevant initial measures will be evaluated with t-tests or chi-square analyses. The effects of the programme on the development of depressive symptoms will be evaluated for the 6-, 12-, and 18-month data using the Cox proportional hazards model to estimate survival curves and rates for different thresholds of combined scores of depressive symptoms. Index parental depression at baseline will be tested as a moderator in the evaluation of the prevention programme effects. Even though the sample in this part of the project is rather small, we will stratify all results by selected measures collected in the baseline questionnaire including gender, socioeconomic status, and mobile/internet use, among others. With regard to the small sample size in this early stage of the study, drop outs or non-completion of outcome measures will be analysed with intent-to-treat.

The qualitative data collected through interviews and focus group discussions will not require any statistical analysis, but they will be analysed using thematic qualitative analytical methods.

Ethics and Dissemination

This study is approved by the Swedish Ethical Review Board (reference number 2019-03347) in Gothenburg. Since participants are adolescents <15 years, consent from both parents (all parents if eligible) and the adolescents themselves will be obtained, as required by applicable laws and regulations.

This study is based on a validated programme that has been conducted in other countries showing good preventive effect. Based on previous studies, we expect preventive effects of the intervention in the participating adolescents. To ensure that the risks are minimal throughout the programme implementation and follow-up, we will provide all participants from the pre-intervention phase

For peer review only

through post-intervention follow-up, in both control and intervention arms, with access to professional help in the form of psychologists, doctors, and nurses if needed. The research group has solid experience implementing the treatment programme in other settings; their experience indicates that the intervention is perceived as positive by students and that participation in the group work has not been stigmatising. All measures and assessments used in the study are validated and tested in many other studies. All researchers and professionals in this study have good paediatric psychiatric competence. They are accustomed to talking with adolescents and will be able to carefully and sensitively handle unpredictable situations. All personnel involved in the study receive training in the intervention. The research team is trained in good clinical practice GCP (scientific quality and ethical standards for planning, conducting, recording and reporting trials involving human participation). The study subjects will never be alone during the intervention, because the intervention is carried out in groups and by trained staff. We will ensure the privacy of study participants, and all relevant legislation will be followed regarding use of research data. All members of the research group have undergone training in the intervention by the founders of the programme. The research group consists of general practitioners', psychologists, specialist nurses, and social workers. The trial manager is a specialist psychologist and a PhD, and the implementation leader is a specialist nurse and PhD. The founders of the programme are both professors of psychology, one in the United States and the other one in Iceland.

Results from the study will be presented so that no individuals, including students, parents, and the employer representatives, will be identifiable. All results will be presented on a group level. All participation is voluntary, and the student can withdraw from the study at any time.

The trial will be conducted in compliance with this study protocol, the Declaration of Helsinki and its modifications, and Good Clinical Practice (GCP).

We plan to disseminate the knowledge gained from this study by publishing our results in peer-reviewed scientific journals, giving presentations at national and international conferences, and spreading information through local and national media channels. We will approach local and national politicians to ensure prolongment of the project.

Discussion

The study protocol of this research project shows that the planning and organisation of the research make it feasible to answer the four research questions.

Question (1). Is the "Thoughts and Health" programme feasible in the context of Swedish schools, from the perspective of the students participating in the programme? The answer will be based on the qualitative interviews and focus group discussions conducted with adolescents participating in the intervention group, as well as analysis of the course surveys gathered after each session and analysis of possible dropouts.

Question (2). Is the "Thoughts and Health" programme feasible in the context of Swedish schools, from the perspective of the primary care professionals leading and supporting the programme implementation? The answer will be based on the analysis of interviews with the professionals who have taken part in individual interviews after the completion of the intervention course, as well as analysis of the course surveys gathered after each session.

Question (3). Does participation in the "Thoughts and Health" programme have an effect on the development of depressive symptoms? The answer will be based on collected data and statistical analyses. Outcomes will be collected before, at and after baseline, based on adolescents' and adults'

For peer review only

reports. Comparisons of effect sizes will be made between the arms of the study (control schools and intervention schools). Measures are described in the protocol and in Table 2.

Question (4). Will participation in the course “Thoughts and Health” increase the participants' chances of completing middle school with high school eligibility? The answer will be based on follow-up measurements of completion and final grades at graduation from middle school. Comparisons of effect sizes will be made between the arms of the study (control schools and intervention schools).

Current trial status

Recruitment of participants started in September 2019, and the last participant is expected to reach the primary endpoint (18-month follow-up) in April 2021. Primary data analysis will begin in May 2021. The naturalistic follow-up phase of the trial will continue until October 2022.

Patient and public involvement

We will receive input from participants and professionals from focus group discussions and interviews, which will guide the continuation of the intervention study. In the current trial, no participants were involved in the design of the study or in the decision of outcome measures.

Members from the public or students were not involved in designing this study.

We will assess the effect of the trial interventions on the participants by collecting information through e-mailed self-rating questionnaires at the end of programme.

For peer review only

Table 1. *Inclusion and exclusion criteria.* *assessed and determined by a psychological assessment, based on the clinical rating instruments CDI, MINI-KID, RCADS and the children's current status

Inclusion criteria	Exclusion criteria
Eighth grader	Ongoing or past depression
Guardian of eighth grader	Addiction
Understand Swedish language skills, written and oral	Past or current suicidal attempt or ideation
Be able to fill out questionnaires independently	Neuropsychiatric diagnosis that precludes group activities
Completed CDI and be above 75e percentile	Not high enough level of function on the CGAS (cut-off ≥ 61)
Completed psychological assessment	Lacking sufficient Swedish language skills, written and oral
Written and oral consent	Serious mental illness*
	Ongoing other psychotherapeutic treatment

Table 2. *Instruments and follow-up assessments. Parents o, adolescents x.*

	Screening	Pre Intervention	During treatment	Post-treatment	6-month follow-up	12-month follow-up	18-month follow-up
**Clinician-rated instruments**			**6 weeks**	**3 months**			
MINI-KID		x		x	x	x	x
CGAS		x		x	x	x	x
CGI				x	x	x	x
**Self-rated instruments**							
CDI	x	x	x	x	x	x	x
RCADS		x, o	x	x	x	x	x
EWSAS		x	x	x	x	x	x
MHC-SF		x	x	x	x	x	x
Baseline Questionnaire		x, o		x, o	x, o	x, o	x, o
**Course surveys**			x	x	x	x	x
**Qualitative method**				**3 months**			
Focus group discussions				x			
Individual interviews				x			

Acknowledgement

We would like to thank participating schools. We would like to acknowledge the people who are participating in making this project real. Thank you School Nurses Helena Nordevik Pedersen, Gunilla Börjesson, and Inger Höber, Psychologists Camilla Johansson, Magnus Hjorth, and Henrik Tomasic, Head of Primary Care Centre in Tjörn Ann-Sofie Lekander, GP Torbjörn Erneholm, and Head of School Health in Tjörn Anna Orvefors. Thanks to Maria EH Larsson.Thanks to Jenna Anderson from Grants Office (Gothia Forum) for help in funding process.

Funding

Social investment grants from Region Västra Götaland, Sweden. Granted: RUN 2018-00614.

Declaration of interest

The author(s) of this paper declare(s) that there is no conflict of interest.

For peer review only

Authors' contributions

All the authors have contributed, are contributing and will contribute to this study as follows: Substantial contributions to the conception or design of the work: CW, JL.L, GS, WEC, IM and EÖA. Planning, conducting and reporting of the work: CW, J.L.L, PA, and G.S, Drafting the manuscript: CW and JL.L. Critical revision of the article: all declared authors. Final approval of the version published: all declared authors. Agreement to be accountable for all aspects of the work: all declared authors.

Figure Legend

Figure 1

Flowchart over the Thought and health study.

For peer review only

References

1. Hankin BL, Abramson LY, Moffitt TE, *et al.* Development of depression from preadolescence to young adulthood: emerging gender differences in a 10-year longitudinal study. *J Abnorm Psychol* 1998;107:128–40. doi:10.1037//0021-843x.107.1.128

2. Lewinsohn PM, Rohde P, Klein DN, *et al.* Natural course of adolescent major depressive disorder: I. Continuity into young adulthood. *J Am Acad Child Adolesc Psychiatry* 1999;38:56–63. doi:10.1097/00004583-199901000-00020

3. Arnarson EO, Craighead WE. Prevention of depression among Icelandic adolescents. *Behav Res Ther* 2009;47:577–85. doi:10.1016/j.brat.2009.03.011

4. Arnarson EO, Craighead WE. Prevention of depression among Icelandic adolescents: a 12-month follow-up. *Behav Res Ther* 2011;49:170–4. doi:10.1016/j.brat.2010.12.008

5. Services S beredning för medicinsk och social utvärdering (SBU); SA for HTA and A of S. Program för att förebygga psykisk ohälsa hos barn. SBU 2010. https://www.sbu.se/sv/publikationer/SBU-utvarderar/program-for-att-forebygga-psykisk-ohalsa-hos-barn/ (accessed 3 Feb 2020).

6. Garber J, Clarke GN, Weersing VR, *et al.* Prevention of depression in at-risk adolescents: a randomized controlled trial. *JAMA* 2009;301:2215–24. doi:10.1001/jama.2009.788

7. Stice E, Rohde P, Seeley JR, *et al.* Brief Cognitive-Behavioral Depression Prevention Program for High-Risk Adolescents Outperforms Two Alternative Interventions: A Randomized Efficacy Trial. *J Consult Clin Psychol* 2008;76:595. doi:10.1037/a0012645

8. Clarke GN, Hawkins W, Murphy M, *et al.* Targeted prevention of unipolar depressive disorder in an at-risk sample of high school adolescents: a randomized trial of a group cognitive intervention. *J Am Acad Child Adolesc Psychiatry* 1995;34:312–21. doi:10.1097/00004583-199503000-00016

9. Erik H. Erikson. Identity, youth and crisis. New York: WW Norton Company, 1968

10. Gillham JE, Hamilton J, Freres DR, et al. Preventing depression among early adolescents in the primary care setting: a randomized controlled study of the Penn Resiliency Program. *J Abnorm Child Psychol* 2006;34:203–19. doi:10.1007/s10802-005-9014-7

11. Seligman MEP, Schulman P, Tryon AM. Group prevention of depression and anxiety symptoms. *Behav Res Ther* 2007;45:1111–26. doi:10.1016/j.brat.2006.09.010

12. Stice E, Burton E, Bearman SK, et al. Randomized trial of a brief depression prevention program: an elusive search for a psychosocial placebo control condition. *Behav Res Ther* 2007;45:863–76. doi:10.1016/j.brat.2006.08.008

13. Horowitz JL, Garber J. The prevention of depressive symptoms in children and adolescents: A meta-analytic review. *J Consult Clin Psychol* 2006;74:401–15. doi:10.1037/0022-006X.74.3.401

14. Merry S, McDowell H, Hetrick S, et al. Psychological and/or educational interventions for the prevention of depression in children and adolescents. *Cochrane Database Syst Rev* 2004;:CD003380. doi:10.1002/14651858.CD003380.pub2

For peer review only

15. Roberts CM, Kane R, Bishop B, et al. The prevention of anxiety and depression in children from disadvantaged schools. *Behav Res Ther* 2010;48:68–73. doi:10.1016/j.brat.2009.09.002

16. Sawyer MG, Pfeiffer S, Spence SH, et al. School-based prevention of depression: a randomised controlled study of the beyondblue schools research initiative. *J Child Psychol Psychiatry* 2010;51:199–209. doi:10.1111/j.1469-7610.2009.02136.x

17. Klein DN, Glenn CR, Kosty DB, *et al.* Predictors of first lifetime onset of major depressive disorder in young adulthood. *J Abnorm Psychol* 2013;122:1–6. doi:10.1037/a0029567

18. Chan A-W, Tetzlaff JM, Altman DG, Laupacis A, Gøtzsche PC, Krleža-Jerić K, Hróbjartsson A, Mann H, Dickersin K, Berlin J, Doré C, Parulekar W, Summerskill W, Groves T, Schulz K, Sox H, Rockhold FW, Rennie D, Moher D. SPIRIT 2013 Statement: Defining standard protocol items for clinical trials. Ann Intern Med. 2013;158(3):200-207

19. Ivarsson T, Svalander P, Litlere O. The Children's Depression Inventory (CDI) as measure of depression in Swedish adolescents. A normative study. *Nordic Journal of Psychiatry*, 60 (3) (2006), pp. 220-226

20. Sheehan, D.V.; Sheehan, K.H.; Shyte, R.D.; Janavs, J.; Bannon, Y.; Rogers, J.E.; Milo, K.M.; Stock, S.L.; Wilkinson, B. Reliability and validity of the Mini International Neurpsychiatric Interview for Children and Adolescents (MINI-KID). *J. Clin. Psychiatry* 2010, 71, 313–326.

21. Chorpita, B. F., Moffitt, C., & Gray, J. (2005). Psychometric properties of the Revised Child Anxiety and Depression Scale in a clinical sample. *Behaviour Research and Therapy*, 43, 309-322.

22. American Psychiatric Association. (2013). Diagnostic and statistical manual of mental disorders (5th ed.). Washington, DC: Author.

23. Shaffer, D., Gould, M.S., Brasic, J., Ambrosini, P., Fisher, P., Bird, H., & Aluwahlia, S. (1983). A Children’s Global Assessment Scale (CGAS). *Archives of General Psychiatry*, 40, 1228-1231.

24. Mundt, J.C., Marks, I.M., Shear K.M., Greist J., (2002). Impairment in functioning. The work and social adjustment scale: a simple measure of impairment of functioning. *The British Journal of Psychiatry*, 180, 461-464.

25. Keyes, C. L. M. (2009). Atlanta: Brief description of the mental health continuum short form (MHC-SF). Available: https://www.aacu.org/sites/default/files/MHC-SFEnglish.pdf. [On–line, retrieved 2020-03-30]

26. Keyes CLM, Wissing M, Potgieter JP, *et al.* Evaluation of the mental health continuum-short form (MHC-SF) in setswana-speaking South Africans. *Clin Psychol Psychother* 2008;15:181–92. doi:10.1002/cpp.572

27. Keyes CLM. The Subjective Well-Being of America’s Youth: Toward a Comprehensive Assessment. *Adolescent & Family Health* 2006;4:3–11.

28. Lamers SMA, Glas CAW, Westerhof GJ, *et al.* Longitudinal evaluation of the mental health continuum-short form (MHC-SF): Measurement invariance across demographics, physical illness, and mental illness. *Eur J Psychol Assess* 2012;28:290–6. doi:10.1027/1015-5759/a000109

For peer review only

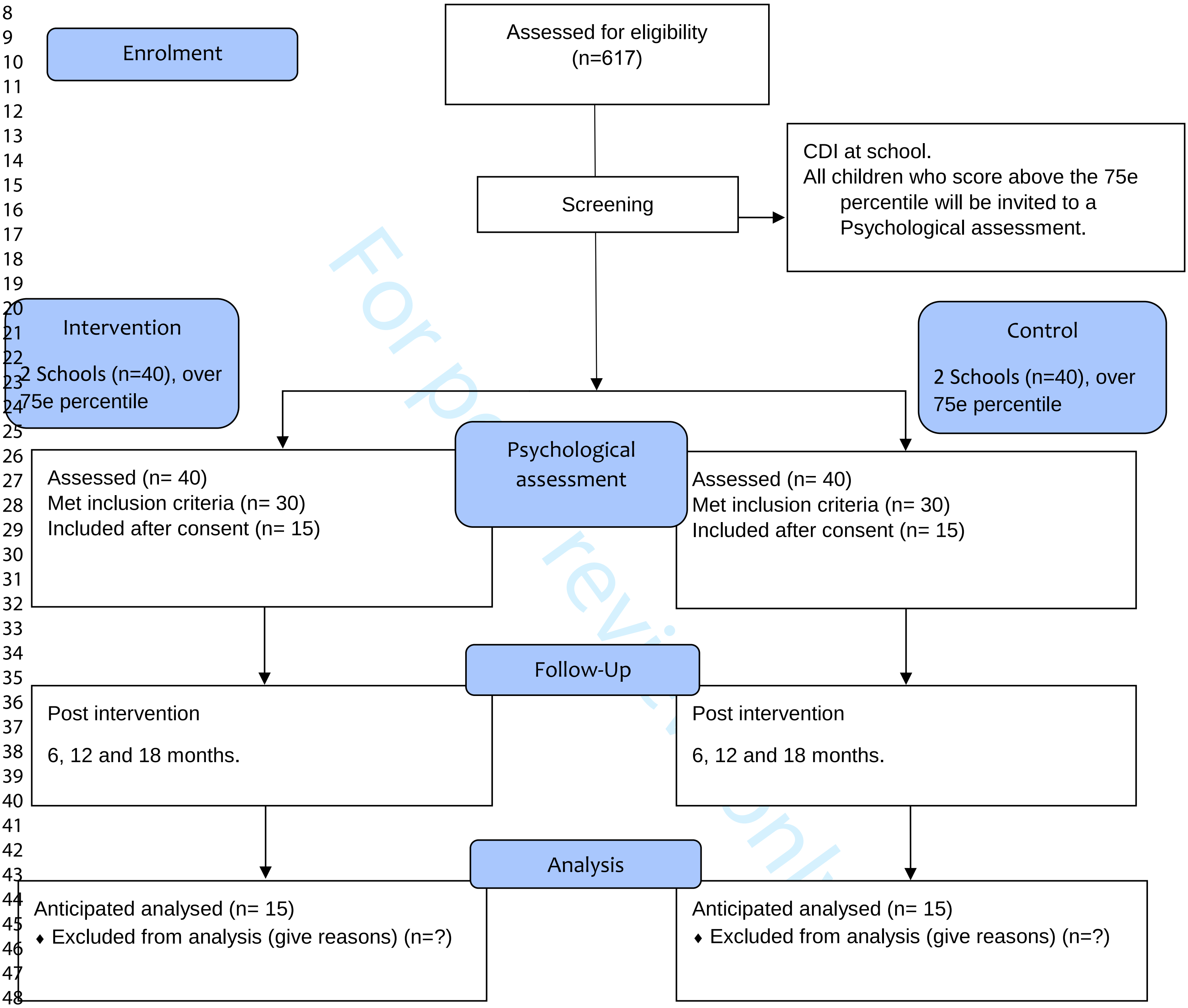
Enrolment
Assessed for eligibility (n=617)
Screening
CDI at school.
All children who score above the 75e percentile will be invited to a Psychological assessment.
Intervention
2 Schools (n=40), over 75e percentile
Control
2 Schools (n=40), over 75e percentile
Psychological assessment
Assessed (n= 40)
Met inclusion criteria (n= 30)
Included after consent (n= 15)
Assessed (n= 40)
Met inclusion criteria (n= 30)
Included after consent (n= 15)
Follow-Up
Post intervention
6, 12 and 18 months.
Post intervention
6, 12 and 18 months.
Analysis
Anticipated analysed (n= 15)
♦ Excluded from analysis (give reasons) (n=?)
Anticipated analysed (n= 15)
♦ Excluded from analysis (give reasons) (n=?)

Information about the study "Thoughts and health" - to prevent mental illness in young people

Background

Mental illness in young people is a growing problem in Sweden and a large part of the world.
Depression is one of the diseases that has the greatest social and economic consequences globally and leads to great human suffering. A first illness often occurs in late adolescence. A first depression increases the risk of relapse.

Preventive efforts to reduce the risk of a first illness should be regarded as an important measure to improve young people's mental health and opportunities for a positive entry into adulthood.

Children with depression at the end of junior high school may risk not being able to finish their compulsory schooling with complete grades, which in turn may affect their future opportunities.

We have now translated and adapted a prevention programme from Icelandic to Swedish. We would like to test whether this programme works in Swedish schools.

In collaboration with Elevhälsan Tjörn Municipality and Orust Municipality and Närhälsan's health centres in the local area and Närhälsan's Research and Development Department, we now want to offer students in these municipalities the opportunity to help us implement the programme and at the same time fill important knowledge gaps in research.

Purpose

To study the effects of an Icelandic prevention programme "Thoughts and health" in Swedish schools.

Request for participation

You are now asked as a student in year 8 on Tjörn for consent to participate in the study and the Thoughts and Health programme.

How is the study done?

All children at the school have been asked to fill out a form with questions about their mental health. All children will receive feedback about the results. Some will receive feedback during their health talk with

the school nurse while others will be offered feedback and assessment by psychologists associated with the project for possible inclusion in the prevention programme. The programme consists of a course involving 12 sessions at the school that are led by a psychologist and the school nurse. The group(s) is put together based on the number of possible participants per school, which means that the composition does not depend upon which class you are in. For you, this means that you will be asked to fill out forms at the start, when half the programme is completed and again at follow-ups. As a participant in the programme, you can of course talk to your parents or guardians about the content of the course, but you should not tell about other participants' experiences. Everyone who is part of the programme or the reference group that is included will be followed up after the end of the programme, at 6, 12 and 18 months. The results from the first questionnaire and the psychologist's assessment are included in the data collected.

Data management and confidentiality

All answers and results will be treated with confidentiality so that unauthorized persons cannot take part in them.
Personal data is registered in accordance with GDPR (The General Data Protection Regulation). The investigator responsible for the study is entrusted with the processing of personal data. According to the GDPR, you have the right to apply for information about which personal data is processed and to demand destruction of the personal data. You and your parents can send your application to the contact person below.

For peer review only

If you want to submit a complaint regarding the processing of personal data, you can contact the Data Inspectorate, which is the supervisory authority for the Data Protection Ordinance. Please note that the Data Protection Officer cannot answer questions concerning specific information about the study but only regarding the application of the Data Protection Regulation.
Contact details of the Data Protection Officer: Anders Larsson, Information Security Coordinator Phone: 070-6552809 anders.h.larsson@vgregion.se
In data processing, your name and personal identity number will be replaced with a code to ensure anonymity. Only those responsible for the study have access to the "code key". Your information will not be passed on. The data will be saved for ten years. The authority responsible for your personal data is primary care Västra Götaland. Lina Kolsmyr tel.
010-441 05 30, narhalsan.dataskyddsombud@vgregion.se Personal data representative Närhälsan, Lillhagsparken 6, 422 50 Hisingsbacka. When the data from the study is compiled, individual individuals will not be able to be identified.

Remuneration
No remuneration will be given for participation.

Volunteering
Your participation is voluntary and you can cancel your participation at any time without giving any reason.

What does the study mean for you and other children in the future?
That you may contribute to the development of student health and that by participating in the research project, you may provide answers to important questions regarding mental health. Your participation will help us to study this prevention programme against depression, the aim of which is to create good routines that in the long run may lead to improved mental health for children and young people.

Persons responsible for the study
If you have any questions, you are welcome to contact us.

Carl Wikberg
PhD, Distriktssjuksköterska
carl.wikberg@allmed.gu.se
0703-821660

Gudny Sveinsdottir
Projektledare, Spec. allmänmedicin
gudny.sveinsdottir@vgregion.se
mobil 0707-980436

Anna Orvefors,
Tf avdelningschef Resurscentrum, Barn- och utbildningsförvaltningen, Tjörns kommun;
anna.orvefors@tjorn.se
tel 0304-601587

Josefine Lilja
FoU Primärvård, Göteborg- och Södra Bohuslän
PhD, Spec. klinisk psykolog, leg. psykoterapeut-KBT, Handledare-KBT
josefine.lilja@vgregion.se
076 - 940 29 69

For peer review only

Consent form - Information about the study "Thoughts and health" - to prevent mental illness in young people

I have been informed, given the opportunity to ask questions, have them answered and agreed to participate in the study and been informed about the right to integrity when the material is published. I have also been informed that my participation in the study is voluntary and that I am right at any time to interrupt my participation in the study without giving a reason and without it affecting my contact with primary care or school.

Print Name of Participant____

Signature of Participant ____

Date ______________________________
Day/month/year

Mailadress____

Mobilenumber____

Print Name of Researcher/person taking the consent__________________________________

Signature of Researcher /person taking the consent___________________________________

Date ______________________________
Day/month/year

For peer review only

Reporting checklist for protocol of a clinical trial.

Based on the SPIRIT guidelines.

Instructions to authors

Complete this checklist by entering the page numbers from your manuscript where readers will find each of the items listed below.

Your article may not currently address all the items on the checklist. Please modify your text to include the missing information. If you are certain that an item does not apply, please write "n/a" and provide a short explanation.

Upload your completed checklist as an extra file when you submit to a journal.

In your methods section, say that you used the SPIRITreporting guidelines, and cite them as:

Chan A-W, Tetzlaff JM, Altman DG, Laupacis A, Gøtzsche PC, Krleža-Jerić K, Hróbjartsson A, Mann H, Dickersin K, Berlin J, Doré C, Parulekar W, Summerskill W, Groves T, Schulz K, Sox H, Rockhold FW, Rennie D, Moher D. SPIRIT 2013 Statement: Defining standard protocol items for clinical trials. Ann Intern Med. 2013;158(3):200-207

		Reporting Item	Page Number
**Administrative information**			
Title	#1	Descriptive title identifying the study design, population, interventions, and, if applicable, trial acronym	1

For peer review only

Trial registration	#2a	Trial identifier and registry name. If not yet registered, name of intended registry	1
Trial registration: data set	#2b	All items from the World Health Organization Trial Registration Data Set	n/a
Protocol version	#3	Date and version identifier	n/a
Funding	#4	Sources and types of financial, material, and other support	11
Roles and responsibilities: contributorship	#5a	Names, affiliations, and roles of protocol contributors	1
Roles and responsibilities: sponsor contact information	#5b	Name and contact information for the trial sponsor	n/a
Roles and responsibilities: sponsor and funder	#5c	Role of study sponsor and funders, if any, in study design; collection, management, analysis, and interpretation of data; writing of the report; and the decision to submit the report for publication, including whether they will have ultimate authority over any of these activities	n/a
Roles and responsibilities: committees	#5d	Composition, roles, and responsibilities of the coordinating centre, steering committee, endpoint adjudication committee, data management team, and	n/a

For peer review only

		other individuals or groups overseeing the trial, if applicable (see Item 21a for data monitoring committee)	
**Introduction**			
Background and rationale	#6a	Description of research question and justification for undertaking the trial, including summary of relevant studies (published and unpublished) examining benefits and harms for each intervention	2
2Background and rationale: choice of comparators	#6b	Explanation for choice of comparators	2
Objectives	#7	Specific objectives or hypotheses	2
Trial design	#8	Description of trial design including type of trial (eg, parallel group, crossover, factorial, single group), allocation ratio, and framework (eg, superiority, equivalence, non-inferiority, exploratory)	3
**Methods: Participants, interventions, and outcomes**			
Study setting	#9	Description of study settings (eg, community clinic, academic hospital) and list of countries where data will be collected. Reference to where list of study sites can be obtained	3

For peer review only

Eligibility criteria	#10	Inclusion and exclusion criteria for participants. If applicable, eligibility criteria for study centres and individuals who will perform the interventions (eg, surgeons, psychotherapists)	3
Interventions: description	#11a	Interventions for each group with sufficient detail to allow replication, including how and when they will be administered	7
Interventions: modifications	#11b	Criteria for discontinuing or modifying allocated interventions for a given trial participant (eg, drug dose change in response to harms, participant request, or improving / worsening disease)	n/a
Interventions: adherance	#11c	Strategies to improve adherence to intervention protocols, and any procedures for monitoring adherence (eg, drug tablet return; laboratory tests)	n/a
Interventions: concomitant care	#11d	Relevant concomitant care and interventions that are permitted or prohibited during the trial	n/a
Outcomes	#12	Primary, secondary, and other outcomes, including the specific measurement variable (eg, systolic blood pressure), analysis metric (eg, change from baseline, final value, time to event), method of aggregation (eg, median, proportion), and time point for each outcome. Explanation of the clinical relevance of chosen efficacy and harm outcomes is strongly recommended	5

For peer review only

Participant timeline	#13	Time schedule of enrolment, interventions (including any run-ins and washouts), assessments, and visits for participants. A schematic diagram is highly recommended (see Figure)	10
Sample size	#14	Estimated number of participants needed to achieve study objectives and how it was determined, including clinical and statistical assumptions supporting any sample size calculations	7
Recruitment	#15	Strategies for achieving adequate participant enrolment to reach target sample size	4
**Methods: Assignment of interventions (for controlled trials)**			
Allocation: sequence generation	#16a	Method of generating the allocation sequence (eg, computer-generated random numbers), and list of any factors for stratification. To reduce predictability of a random sequence, details of any planned restriction (eg, blocking) should be provided in a separate document that is unavailable to those who enrol participants or assign interventions	n/a
Allocation concealment mechanism	#16b	Mechanism of implementing the allocation sequence (eg, central telephone; sequentially numbered, opaque,	n/a

For peer review only

		sealed envelopes), describing any steps to conceal the sequence until interventions are assigned	
Allocation: implementation	#16c	Who will generate the allocation sequence, who will enrol participants, and who will assign participants to interventions	n/a
Blinding (masking)	#17a	Who will be blinded after assignment to interventions (eg, trial participants, care providers, outcome assessors, data analysts), and how	n/a
Blinding (masking): emergency unblinding	#17b	If blinded, circumstances under which unblinding is permissible, and procedure for revealing a participant's allocated intervention during the trial	n/a
**Methods: Data collection, management, and analysis**			
Data collection plan	#18a	Plans for assessment and collection of outcome, baseline, and other trial data, including any related processes to promote data quality (eg, duplicate measurements, training of assessors) and a description of study instruments (eg, questionnaires, laboratory tests) along with their reliability and validity, if known. Reference to where data collection forms can be found, if not in the protocol	10

For peer review only

Data collection plan: retention	#18b	Plans to promote participant retention and complete follow-up, including list of any outcome data to be collected for participants who discontinue or deviate from intervention protocols	n/a
Data management	#19	Plans for data entry, coding, security, and storage, including any related processes to promote data quality (eg, double data entry; range checks for data values). Reference to where details of data management procedures can be found, if not in the protocol	7
Statistics: outcomes	#20a	Statistical methods for analysing primary and secondary outcomes. Reference to where other details of the statistical analysis plan can be found, if not in the protocol	7
Statistics: additional analyses	#20b	Methods for any additional analyses (eg, subgroup and adjusted analyses)	n/a
Statistics: analysis population and missing data	#20c	Definition of analysis population relating to protocol non-adherence (eg, as randomised analysis), and any statistical methods to handle missing data (eg, multiple imputation)	n/a
**Methods: Monitoring**			
Data monitoring: formal committee	#21a	Composition of data monitoring committee (DMC); summary of its role and reporting structure; statement of whether it is independent from the sponsor and	n/a

For peer review only

		competing interests; and reference to where further details about its charter can be found, if not in the protocol. Alternatively, an explanation of why a DMC is not needed	
Data monitoring: interim analysis	#21b	Description of any interim analyses and stopping guidelines, including who will have access to these interim results and make the final decision to terminate the trial	n/a
Harms	#22	Plans for collecting, assessing, reporting, and managing solicited and spontaneously reported adverse events and other unintended effects of trial interventions or trial conduct	8
Auditing	#23	Frequency and procedures for auditing trial conduct, if any, and whether the process will be independent from investigators and the sponsor	n/a
**Ethics and dissemination**			
Research ethics approval	#24	Plans for seeking research ethics committee / institutional review board (REC / IRB) approval	8
Protocol amendments	#25	Plans for communicating important protocol modifications (eg, changes to eligibility criteria, outcomes, analyses) to relevant parties (eg, investigators, REC / IRBs, trial participants, trial registries, journals, regulators)	n/a

For peer review only

Consent or assent	#26a	Who will obtain informed consent or assent from potential trial participants or authorised surrogates, and how (see Item 32)	8
Consent or assent: ancillary studies	#26b	Additional consent provisions for collection and use of participant data and biological specimens in ancillary studies, if applicable	n/a
Confidentiality	#27	How personal information about potential and enrolled participants will be collected, shared, and maintained in order to protect confidentiality before, during, and after the trial	n/a
Declaration of interests	#28	Financial and other competing interests for principal investigators for the overall trial and each study site	11
Data access	#29	Statement of who will have access to the final trial dataset, and disclosure of contractual agreements that limit such access for investigators	7
Ancillary and post trial care	#30	Provisions, if any, for ancillary and post-trial care, and for compensation to those who suffer harm from trial participation	
Dissemination policy: trial results	#31a	Plans for investigators and sponsor to communicate trial results to participants, healthcare professionals, the public, and other relevant groups (eg, via publication, reporting in results databases, or other data sharing arrangements), including any publication restrictions	n/a

For peer review only

Dissemination policy: authorship	#31b	Authorship eligibility guidelines and any intended use of professional writers	n/a
Dissemination policy: reproducible research	#31c	Plans, if any, for granting public access to the full protocol, participant-level dataset, and statistical code	n/a

Appendices

Informed consent materials	#32	Model consent form and other related documentation given to participants and authorised surrogates	attached
Biological specimens	#33	Plans for collection, laboratory evaluation, and storage of biological specimens for genetic or molecular analysis in the current trial and for future use in ancillary studies, if applicable	n/a

None The SPIRIT checklist is distributed under the terms of the Creative Commons Attribution License CC-BY-ND 3.0. This checklist can be completed online using https://www.goodreports.org/, a tool made by the EQUATOR Network in collaboration with Penelope.ai

For peer review only
